# A paradigm shift toward explainable and federated intelligence for brain tumor MRI analysis

**DOI:** 10.3389/frai.2026.1959004

**Published:** 2026-10-22

**Authors:** B. Uthra, R. Sivashankari

**Affiliations:** School of Computer Science Engineering and Information Systems, Vellore Institute of Technology, Vellore, Tamil Nadu, India

**Keywords:** brain tumor analysis, explainable AI (XAI), Federated Learning, trustworthy AI, vision transformers

## Abstract

Deep learning techniques in brain tumor analysis using magnetic resonance imaging (MRI) scans have improved significantly, but their clinical deployment is limited by models with limited interpretability (‘black-box' models) and data privacy limitations. This paper analyses recent literature and aims to explore the intersection of Explainable Artificial Intelligence (XAI) and Federated Learning (FL). Following the PRISMA protocol, a systematic search was conducted in Scopus on February 24, 2026, covering English-language journal articles and conference proceedings published from January 2020 to February 2026. The search initially identified 767 records, which were screened using predefined inclusion and exclusion criteria for MRI-based brain tumor studies addressing deep learning, explainability, privacy, or Federated Learning, resulting in 147 initially retained studies for the final screening set. One study was subsequently identified as formally retracted and excluded from the final synthesis, resulting in 146 included studies. The review classifies the literature into five primary research themes in brain tumor analysis: explainability, deep learning frameworks, privacy-preserving learning, advanced architectures (Transformers and Mamba), and multimodal learning. The literature suggests a paradigm shift in brain tumor analysis toward model interpretability using XAI. However, there is a significant knowledge gap in privacy-preserving collaborative learning models. Advanced architectures are proposed to solve brain tumor problems, but there are still limitations in model generalization, computational costs, and clinical reliability.

## 1 Introduction

Despite achieving high performance on benchmark datasets in brain tumor segmentation and classification, many deep learning (DL)-based neuro-oncology systems remain insufficiently validated for routine clinical deployment. The major barriers limiting clinical translation include poor interpretability, weak external generalization, dependence on centralized datasets, and increasing concerns regarding patient data privacy under regulations such as GDPR and HIPAA. Consequently, recent neuro-oncology research has gradually shifted from performance-oriented AI toward the development of *trustworthy AI* frameworks capable of ensuring transparency, robustness, privacy preservation, and clinically reliable decision support.

Explainable AI (XAI) has emerged as a critical component of trustworthy neuro-oncology systems because clinicians require biologically meaningful reasoning rather than predictions from black-box models, whose internal decision processes are not readily interpretable to clinicians. In brain tumor analysis, XAI methods such as Gradient-weighted Class Activation Mapping (Grad-CAM), SHapley Additive exPlanations (SHAP), and saliency-based visualization are increasingly used to identify whether neural networks focus on clinically relevant pathological regions such as enhancing tumor cores, necrosis, and peritumoral edema instead of imaging artifacts. Simultaneously, Federated Learning (FL) has gained considerable attention as a privacy-preserving collaborative learning paradigm that enables multi-institutional magnetic resonance imaging (MRI) analysis without direct data sharing. The convergence of XAI and FL therefore represents an important step toward secure, transparent, and clinically adaptive neuro-oncology AI systems. In parallel, recent advances in architectural design have accelerated the evolution of brain tumor analysis frameworks from conventional neural network-based models toward Vision Transformers (ViTs) (Dosovitskiy, 2020), hybrid attention-driven systems, and State Space Models (SSMs) such as Mamba, which are capable of modeling long-range spatial dependencies in high-dimensional multi-modal MRI volumes (Gu and Dao, 2023). Moreover, the increasing integration of multimodal MRI, radiogenomics, digital pathology, and clinical metadata has expanded the role of AI beyond tumor detection toward prognosis prediction, treatment planning, and personalized neuro-oncology.

Although numerous review articles have independently discussed deep learning, explainability, Federated Learning, or multimodal neuroimaging, a focused synthesis connecting these domains under the broader perspective of trustworthy neuro-oncology AI remains limited. In particular, there is still insufficient critical understanding regarding the tradeoffs between interpretability, privacy preservation, computational scalability, multimodal fusion, and real-world clinical deployment.

### 1.1 Research gap and open problems

Despite substantial progress in brain tumor MRI analysis, the reviewed literature reveals several specific open research problems that remain insufficiently addressed. First, explainability is predominantly implemented through *post-hoc* visualization methods such as Grad-CAM, SHAP, and saliency maps, while quantitative evaluation of explanation reliability, biological plausibility, and agreement with clinical experts remains limited. Second, XAI and Federated Learning are rarely integrated into a unified framework, leaving a methodological gap between interpretable prediction and privacy-preserving multi-institutional learning. Third, federated approaches remain challenged by non-independent and non-identically distributed (non-IID) data arising from differences in scanners, acquisition protocols, patient populations, missing modalities, and annotation practices, while privacy-preserving mechanisms may introduce additional performance degradation and communication overhead. Fourth, the strong dependence on benchmark datasets limits assessment of robustness under real-world domain shifts, external institutions, and heterogeneous clinical conditions. Fifth, multimodal and multi-omics approaches remain computationally demanding and are not yet consistently evaluated for calibration, interpretability, and clinical usefulness.

These gaps motivate future XAI-FL frameworks that jointly address explanation validity, privacy preservation, heterogeneous data distributions, and clinical robustness. Such frameworks could incorporate quantitatively evaluated XAI with clinician-informed and biologically grounded explanations, adaptive federated optimization for non-IID clients, privacy mechanisms with explicit utility monitoring, multimodal fusion capable of handling missing modalities, and standardized evaluation protocols covering predictive performance, calibration, uncertainty, explanation quality, robustness, and clinical utility. The resulting research direction is therefore not simply to improve predictive accuracy, but to develop integrated and externally validated trustworthy AI systems capable of supporting privacy-preserving and clinically interpretable brain tumor analysis across institutions. Hence, the current survey provides a focused systematic synthesis of 146 studies from 2020 to 2026 related to trustworthy AI for brain tumor MRI analysis. The contributions made by this systematic review can be listed as follows:

- Thematic synthesis of deep learning, explainable AI, Federated Learning, multimodal integration, and clinically adaptive neuro-oncology approaches.
- A focused cross-paradigm evaluation of CNNs, transformers, Mamba neural networks, and hybrid optimization-based systems considering interpretability, robustness, privacy-preserving, and clinical reliability aspects.
- Detailed analysis of translational barriers that hamper real-world applications such as data dependence, insufficient external validation, limited explainability, computational complexity, and regulatory issues.
- A holistic view on future trustworthy AI systems for neuro-oncology, integrating explanations, federated intelligence, multimodal reasoning, and clinical validation approaches.

The rest of the paper is outlined as follows. In Section II, the background and preliminaries of trustworthy AI-based brain tumor detection using technologies such as explainable AI, Federated Learning, and multimodal MRI analysis are presented. Section III outlines the method for literature review, specifically the study selection criteria following the PRISMA guidelines and the thematic evaluation criteria adopted for this survey. In Section IV, a detailed thematic analysis is provided about deep learning approaches for brain tumor analysis including CNN, transfer learning, explainable AI, multimodal/radiomics frameworks, robust learning techniques, workflow-based clinical solutions, and optimization-based neuro-oncology solutions. In Section V, we discuss some of the major barriers to the adoption of trustworthy AI-based brain tumor detection, whereas emerging research directions are discussed in Section VI.

## 2 Background and preliminaries

Moving away from experimental AI-based models to the development of trustworthy real-time neuro-oncology tools necessitates a paradigm change in the approach to learning from accuracy-oriented machine learning to *trustworthy AI*. In terms of clinical applications of brain tumor prediction using images, trustworthy AI not only refers to predictive capability but also involves aspects of explainability, robustness, privacy protection, uncertainty quantification, and adaptability to heterogeneous imaging settings. In this review, clinical reliability refers to the ability of an AI system to provide consistent, robust, and clinically useful predictions under relevant variations in patients, imaging conditions, and clinical settings. Clinical deployment refers to the integration and use of such AI systems within routine clinical workflows for diagnostic, prognostic, or decision-support purposes. Black-box models refer to models whose internal decision processes are not readily interpretable to clinicians.

### 2.1 Explainable AI (XAI)

Conventional deep learning models often operate as black-box models, limiting clinical confidence despite achieving high predictive performance. In brain tumor segmentation and classification, XAI methods such as Gradient-weighted Class Activation Mapping (Grad-CAM) (Selvaraju et al., 2017), SHapley Additive exPlanations (SHAP) (Lundberg and Lee, 2017), Local Interpretable Model-agnostic Explanations (LIME) (Ribeiro et al., 2016), and saliency mapping are increasingly used to associate model activations with anatomically meaningful tumor regions. Such explainability mechanisms are particularly important for differentiating true tumor progression from treatment-related pseudoprogression and for improving clinician trust in automated neuro-oncology systems. However, many current explainability approaches remain *post-hoc* visual approximations without biologically validated causal interpretation. To maintain conceptual consistency throughout the review, explainability-related methods are distinguished according to the type of information they provide and the degree of validation they receive. *Feature attribution* refers to methods that estimate the contribution or importance of input features to a model prediction, including SHAP and LIME. *Saliency visualization* refers specifically to spatial or feature-level visual representations of model sensitivity, such as Grad-CAM, CAM, saliency maps, and attention maps. These visualizations indicate regions or features associated with a prediction but do not, by themselves, establish that the highlighted regions are biologically causal. *Intrinsic interpretability* refers to models whose decision mechanism is designed to be interpretable as part of the model architecture or learning process, rather than applying an explanation after prediction. In contrast, *post-hoc explanation* refers to methods applied after a model has generated a prediction to approximate or visualize the factors associated with that prediction. Grad-CAM, SHAP, LIME, and conventional saliency-based methods are therefore treated primarily as *post-hoc* explanation or attribution approaches unless the reviewed study explicitly integrates them into the learning process. *Counterfactual explanation* considers how a prediction would change under a specified change in the input or relevant feature, thereby providing an alternative form of explanation based on hypothetical modifications. *Causal explanation* requires stronger evidence than attribution or visualization because it seeks to establish whether a feature or biological characteristic has a causal influence on the prediction, typically requiring an intervention-based, mechanistic, or otherwise causally grounded evaluation. Consequently, an attention map, saliency map, or feature-attribution score is not interpreted as evidence of biological causality unless such causal validation is explicitly demonstrated. Finally, *clinician-validated explanation* refers to explanations that have been evaluated by domain experts, such as radiologists, pathologists, or neuro-oncology specialists, for clinical relevance, anatomical plausibility, or agreement with established diagnostic evidence. Clinical trust is treated separately from these explanation types because it represents a broader outcome or property of an AI system rather than a specific explanation mechanism. Similarly, visualization describes the presentation format of an explanation and should not be treated as synonymous with explainability or interpretability.

Accordingly, throughout this review, XAI methods are described using the most specific applicable category: feature attribution, saliency visualization, intrinsic interpretability, *post-hoc* explanation, counterfactual explanation, causal explanation, or clinician-validated explanation. This taxonomy is used to distinguish what an explanation method computes from how the explanation is presented and whether its clinical or biological validity has been demonstrated.

### 2.2 Federated Learning (FL)

Large-scale centralized collection of MRI data is generally hindered by stringent healthcare data protection policies such as General Data Protection Regulation (GDPR) and Health Insurance Portability and Accountability Act (HIPAA). Federated Learning provides an opportunity for collaborative learning in multiple institutions through sharing model updates rather than patient images. In such a decentralized approach, access to diverse neuroimaging data is ensured without compromising data ownership at institutions. However, federated approaches are still plagued by several problems of non-IID (non-independent and identically distributed) data distribution, communication costs, utility-privacy trade-offs, and robustness issues in a heterogeneous clinical setting. Decentralized privacy-preserving techniques and interpretability of AI models in clinical context is theoretically represented in Figure 1.

**Figure 1:**
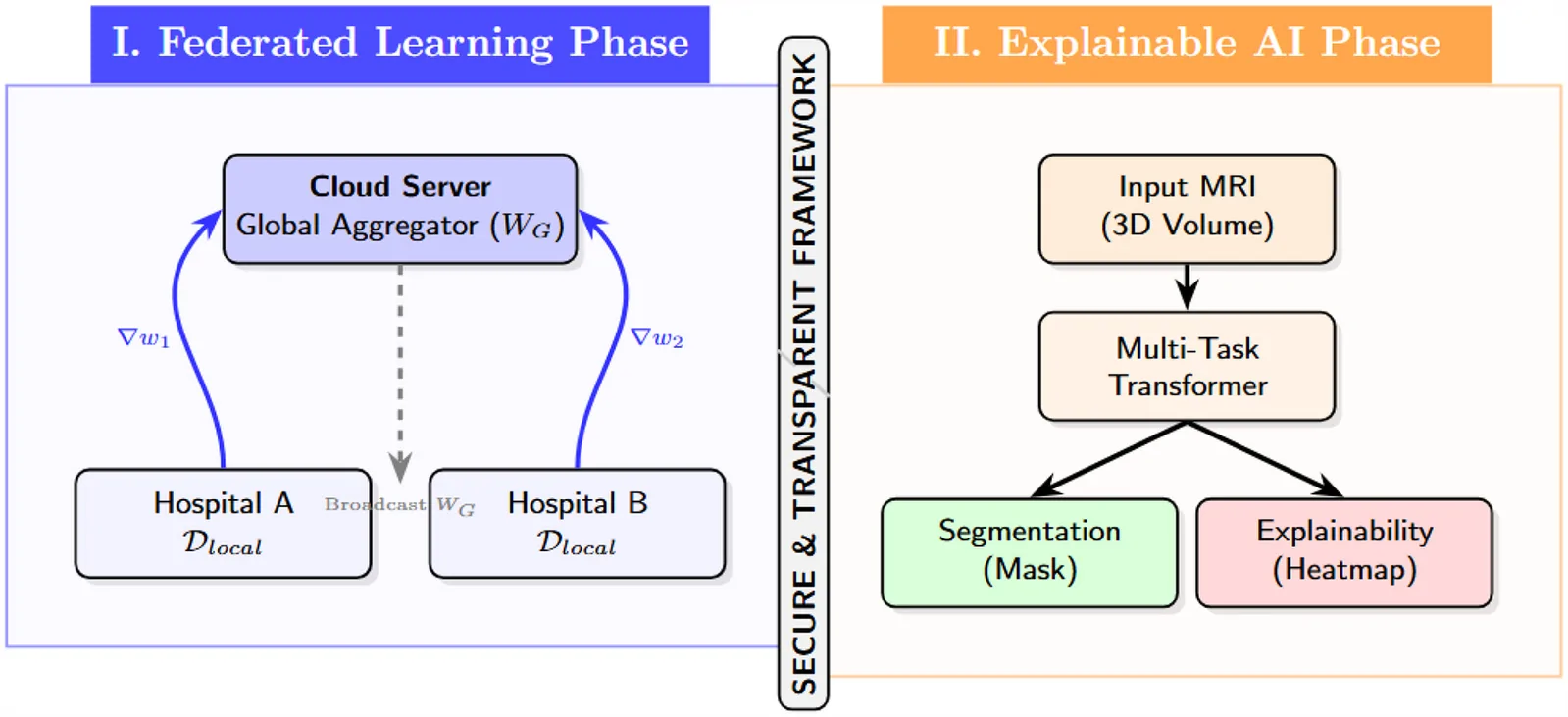
A unified framework for brain tumor analysis that bridges the gap between decentralized data privacy and human-centric model transparency.

### 2.3 Synergy of multi-modal MRI sequences

Multimodality is an intrinsic quality of brain tumor analysis with MRI. For example, T1-weighted (T1), T1 contrast-enhanced (T1c), T2-weighted (T2), and fluid attenuated inversion recovery (FLAIR) images are indispensable in order to have an adequate characterization of a tumor. More specifically, FLAIR is more prone to detecting peritumoral edema, whereas T1c images are more informative in identifying the core and necrosis of enhancing tumors. Thus, contemporary trustworthy neuro-oncology systems tend to incorporate image fusion approaches into their algorithms.

## 3 Methodology

This systematic literature review (SLR) was undertaken by adhering to the guidelines of the *Preferred Reporting Items for Systematic Reviews and Meta-Analyses* (PRISMA) approach in order to ensure methodological transparency and reproducibility (Page et al., 2021). The main aim of this review was to explore recent advancements in brain tumor MRI analysis across five primary research themes: (i) Explainable AI (XAI), (ii) Deep Learning Frameworks, (iii) Privacy and Federated Learning, (iv) Advanced Architectures (Transformers/Mamba), and (v) Multi-modal MRI Integration. The process of literature search entailed several stages including database searching, filtering based on metadata, logical screening and manual verification of articles. All retained studies were manually reviewed to confirm thematic relevance and methodological alignment with the objectives of trustworthy brain tumor detection using AI systems.

### 3.1 Research questions

To guide the systematic synthesis, this review addresses three research questions:

- RQ1: What are the major methodological trends in brain tumor MRI analysis using deep learning, Explainable AI (XAI), Federated Learning (FL), advanced architectures, and multimodal approaches?
- RQ2: How are explainability and privacy-preserving learning addressed in the reviewed literature, and to what extent are XAI and FL integrated within the same brain tumor MRI analysis frameworks?
- RQ3: What are the major limitations and research gaps concerning interpretability, privacy, data heterogeneity, validation, evaluation, and clinical applicability of trustworthy AI for brain tumor MRI analysis?

The intersection of XAI and FL is examined by distinguishing studies that address XAI and FL separately from studies that incorporate both components within the same framework. For studies involving both components, the review considers how interpretability and privacy-preserving collaborative learning are jointly addressed, together with the reported challenges related to data heterogeneity, robustness, and clinical validation.

### 3.2 Search strategy and data sources

The literature search was conducted exclusively in the Scopus database because of its broad multidisciplinary coverage of peer-reviewed journal articles and conference proceedings relevant to medical imaging and artificial intelligence research. The literature search was carried out on February 24, 2026, with respect to the articles published between January 2020 and February 2026. While all articles since 2020 have been considered to reflect their fundamental contributions, analytical focus was made on novel methods proposed during 2024–2026. The search query has included the following three main components: (i) brain tumor pathogenesis, (ii) deep learning networks, and (iii) explainable/trustworthy AI approaches that ensure privacy. The Boolean search string below was applied to the TITLE-ABS-KEY search field:

("Brain Tumor" OR "Glioma" OR "Meningioma") AND ("Deep Learning" OR "Vision Transformer" OR "CNN") AND ("Explainable AI" OR "XAI" OR "Interpretability" OR "Federated Learning" OR "Privacy-Preserving")

The search was restricted to English-language journal articles and conference proceedings with final publication status. No additional bibliographic databases were searched. Scopus was selected because of its broad multidisciplinary coverage of peer-reviewed journal articles and conference proceedings relevant to artificial intelligence, medical imaging, and healthcare research. The database, search date, search field, publication-period restriction, language restriction, document types, and final publication-status filter are reported to facilitate reproducibility.

The search strategy was intentionally designed to identify literature at the intersection of brain tumor MRI analysis, deep learning, and trustworthy AI-related themes, particularly explainability, interpretability, privacy preservation, and Federated Learning. However, requiring these terms in the TITLE-ABS-KEY search may exclude relevant brain tumor deep-learning studies that do not explicitly mention explainability, interpretability, Federated Learning, or privacy in their titles or abstracts. This represents a potential search-induced selection bias. Therefore, the review is not intended to constitute a comprehensive census of all brain tumor AI research; rather, it provides a focused systematic synthesis of literature addressing trustworthy AI-related dimensions of brain tumor MRI analysis. This limitation is considered when interpreting the quantitative trends, thematic distributions, and research gaps identified in the review.

### 3.3 Screening and eligibility criteria

To maintain the technical and clinical significance of this research, a multistep screening process was used. First, using the Scopus search algorithm, 767 documents were retrieved. Further, the screening process continued through various steps for narrowing down the list of candidate studies. The details of the screening process and the number of records at each step are shown in Table 1.

**Table 1:** Multi-stage literature screening pipeline.

Screening stage	Remaining records	Records excluded at this stage
Initial TITLE-ABS-KEY query results	767	–
Time filter (2020–2026)	757	10
Restricted to journal articles and conference proceedings	359	398
English-language publications	358	1
Final publication stage filtering	348	10
Automated logical screening and manual validation	147	201
Post-screening retraction verification	146	1
Final included studies	146	–

The screening counts were reconciled at each sequential stage: 767 records were reduced to 757 after the time-period filter, 359 after document-type restriction, 358 after the English-language restriction, and 348 after final publication-status filtering; automated logical screening and manual validation then reduced the records to 147, followed by exclusion of one formally retracted study, resulting in 146 final included studies. The inclusion and exclusion criteria used during the screening process are summarized in Table 2.

**Table 2:** Inclusion and exclusion criteria.

Inclusion criteria	Exclusion criteria
MRI-based brain tumor studies (T1, T2, T1ce, FLAIR)	Non-MRI modalities (CT, PET, SPECT)
Brain tumor classification, segmentation, or diagnosis	Generic deep learning studies lacking clinical relevance
Studies involving XAI, interpretability, privacy, or Federated Learning	Black-box models without interpretability/privacy discussion
Original journal articles and conference proceedings	Reviews, editorials, book chapters, posters, and non-English publications
Methodological contributions in advanced architectures or trustworthy AI	Incomplete or unpublished records

In order to enhance consistency in terms of methodology, duplicated studies were identified and excluded by means of comparing the DOI and matching titles and metadata. The main screening and study selection procedure was carried out by the lead author in the very beginning by selecting those papers whose titles, abstracts, and methodological aspects fit. Afterwards, these papers were validated by the co-author in order to ensure their relevance and thematic eligibility. If there were any doubts about inclusion/exclusion of a paper, both authors discussed the issue. During post-screening verification, one study, Soni et al. (2024) was identified as formally retracted. The retraction status was verified against the publisher's Wiley Online Library record, and the study was excluded from the final synthesis, resulting in 146 included studies.

### 3.4 Automated logical filtering framework

After filtering through metadata screening, an automated screening process based on logic using Python, was created to increase thematic specificity and exclude studies that are not specialized enough in explainability or privacy-preserving themes. The logical filtering stage retained studies only when:

1. At least one brain tumor pathology-related keyword was detected, and
2. At least one advanced methodological or trustworthy AI keyword was simultaneously present.

The pathology-related keywords included: “Glioma, Glioblastoma, Meningioma, Pituitary, Metastasis, BrATS.” The methodological depth keywords included: “Explainable AI, XAI, Interpretability, Federated Learning, Privacy, Decentralized Learning, Grad-CAM, Saliency, Differential Privacy, Trustworthy AI, Integrated Gradients, Mamba, and Uncertainty Quantification.” The filtering logic operated on publication titles and abstracts using a Boolean co-occurrence strategy: “(Pathology Keyword) AND (Methodological Depth Keyword).” Terms like “CNN” or “Attention,” which are too general for use as filters, were not independently employed for their screening purposes in an attempt to minimize the identification of articles that did not directly address topics related to brain tumor detection. Such filtering favored those studies that tackled not only methodology but also clinically interpretable and privacy-preserving AI algorithms. Even though the automated process ensured more thematic accuracy, studies that had a different semantic meaning for a certain keyword were likely excluded from the screening process due to keyword dependency.

To ensure methodological consistency during literature synthesis, the included studies were comparatively evaluated across four key methodological dimensions: dataset diversity, validation strategy, explainability, and privacy preservation. These dimensions were selected because they directly influence the reliability, clinical applicability, and translational potential of AI-based brain tumor MRI analysis. Rather than assigning formal quality scores, this framework was used to facilitate a structured comparison of methodological characteristics, identify recurring research patterns, and highlight common strengths and limitations across the reviewed literature.

Overall, the thematic evaluation framework (Table 3) provided a structured basis for comparing methodological characteristics across the reviewed studies. It facilitated the identification of dominant research trends, recurring methodological limitations, emerging trustworthy AI strategies, and unresolved challenges in brain tumor MRI analysis while supporting the critical synthesis presented throughout this review.

**Table 3:** Thematic evaluation framework used for literature evaluation.

Criterion	Evaluation focus
Dataset diversity	Single-center vs. multi-center datasets, modality diversity, and MRI sequence heterogeneity
Validation strategy	Internal validation, cross-validation, independent testing, and external clinical validation
Explainability depth	Visual saliency methods vs. quantitatively validated explainability frameworks
Privacy robustness	Federated Learning implementation, differential privacy integration, and decentralized training capability

#### 3.4.1 Operational criteria for qualitative comparative ratings

To ensure consistency and reproducibility in the comparative tables, qualitative ratings were assigned using predefined operational criteria rather than subjective overall impressions. The symbols ✓, △, and × denote strong/full support, partial or limited support, and absent, weak, or insufficient evidence, respectively. The assessment was based on information explicitly reported in the reviewed studies, including the experimental design, validation strategy, datasets, explainability methods, architectural characteristics, and reported deployment considerations. A rating was not assigned as strong when the required evidence was not explicitly reported.

The qualitative coding was performed at the study level by the lead author through structured examination of the full text, tables, and reported experimental methodology. The coding was subsequently checked by the co-author for consistency with the predefined criteria. When the evidence was ambiguous or a rating was disputed, the study was discussed jointly and the rating was assigned according to the reported evidence rather than inferred model capability. Disagreements were resolved through consensus discussion between the authors. No formal inter-rater reliability statistic was calculated, as the coding was not performed as an independent parallel assessment. This absence of formal inter-rater reliability measurement is acknowledged as a limitation of the qualitative comparative assessment. Consequently, the qualitative ratings represent structured evidence-based assessments of reported characteristics and should not be interpreted as formal quality scores or independent clinical-readiness assessments.

### 3.5 Quantitative trend and gap analysis

Trend analysis in terms of the technical focus was subsequently carried out using the corpus finalized during this study to examine the prominent technical priorities, architectural approaches adopted, and potential research directions that may emerge in the field of brain tumor detection using AI system development. The thematic categories used in this review were derived from the recurring methodological directions identified during the screening and full-text analysis of the included studies rather than being imposed as a predefined classification framework. During literature synthesis, studies were examined according to their primary methodological contribution and recurring research focus. This process revealed five dominant themes: Explainable AI (XAI), Deep Learning Frameworks, Privacy and Federated Learning, Advanced Architectures (Transformers/Mamba), and Multi-modal MRI Integration. These themes were subsequently formalized as the primary categories for quantitative trend and gap analysis. Each study was assigned to one primary theme according to its dominant methodological focus to maintain a mutually exclusive classification and ensure consistent quantitative analysis. To ensure reproducibility, explicit operational coding rules were applied during this assignment. Explainable AI (XAI) was assigned when the principal methodological contribution involved explicit explainability or interpretability mechanisms, such as SHAP, LIME, Grad-CAM, saliency-based visualization, intrinsic interpretability, or other explanation mechanisms. Deep Learning Frameworks was assigned when the major contribution revolves around deep learning techniques either traditional or specialized like convolutional neural networks, transfer learning, or hybrid convolutional neural network models, without having another theme to reflect the major methodological contribution. Privacy and Federated Learning was used when Federated Learning or other privacy-based learning methods become the major methodological contribution. Advanced Architectures (Transformers/Mamba) was assigned when Transformer-based, Vision Transformer, Mamba, or related state-space architectures represented the central architectural contribution. Multi-modal MRI Integration was assigned when explicit integration or fusion of multiple MRI sequences and/or complementary clinical, radiomic, genomic, or pathological information constituted the central methodological contribution. When a study contained characteristics associated with more than one theme, it was not counted in multiple categories. Instead, the primary theme was determined from the study's stated objective, methodological novelty, and dominant contribution. For example, a study combining a Transformer architecture, SHAP-based explanation, and multimodal MRI could satisfy characteristics of multiple themes; however, only the theme corresponding to its dominant methodological contribution was recorded for the quantitative analysis. Clinical workflow, prognostic, hybrid, radiomics, and optimization-driven approaches were treated as secondary descriptive paradigms in the qualitative synthesis and were not introduced as additional quantitative categories. Ambiguous cases were resolved through discussion between the authors based on the methodological evidence reported in the full text. The reviewed studies were analyzed across five primary research themes:

- Explainable AI (XAI).
- Deep Learning Frameworks,
- Privacy and Federated Learning,
- Advanced Architectures (Transformers/Mamba),
- Multi-modal MRI Integration.

Specifically, the research showed a high amount of interest in approaches related to explainability and visualization-based interpretation, whereas privacy-enhancing and decentralized learning algorithms were less explored within the domain. Such a finding is crucial in view of the significant challenge posed to clinical AI systems in which rapid progress was made in interpreting results, but large-scale multi-institutional learning has not become widespread due to data privacy and sovereignty concerns. Moreover, multi-modal fusion methods based on T1, T2, T1ce, and FLAIR scans have become increasingly common in the papers aimed at identifying clinical biomarkers of tumors, whereas Vision Transformer and state-space approaches developed using Mamba were gaining momentum in 2024–2026. The distribution of the reviewed studies across the identified thematic categories is illustrated in Figure 2.

**Figure 2:**
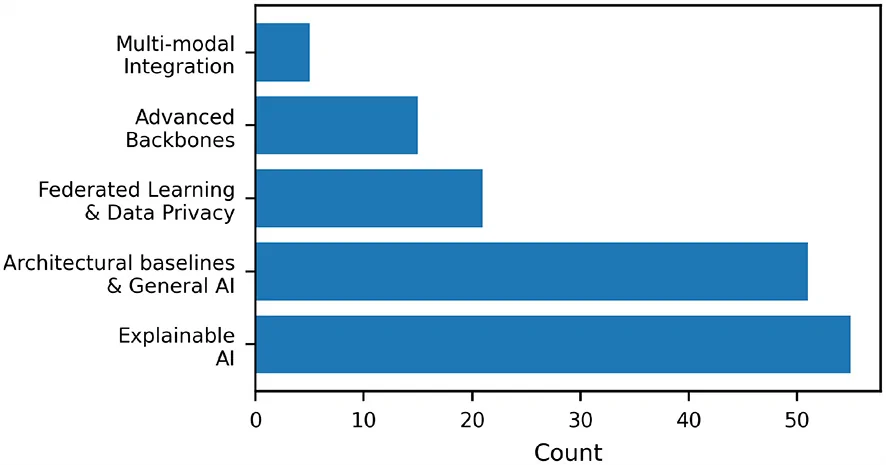
Distribution of reviewed studies across major thematic categories, highlighting the dominance of explainability-focused research and the comparatively limited adoption of privacy-preserving frameworks.

The entire process of literature selection is illustrated in Figure 3.

**Figure 3:**
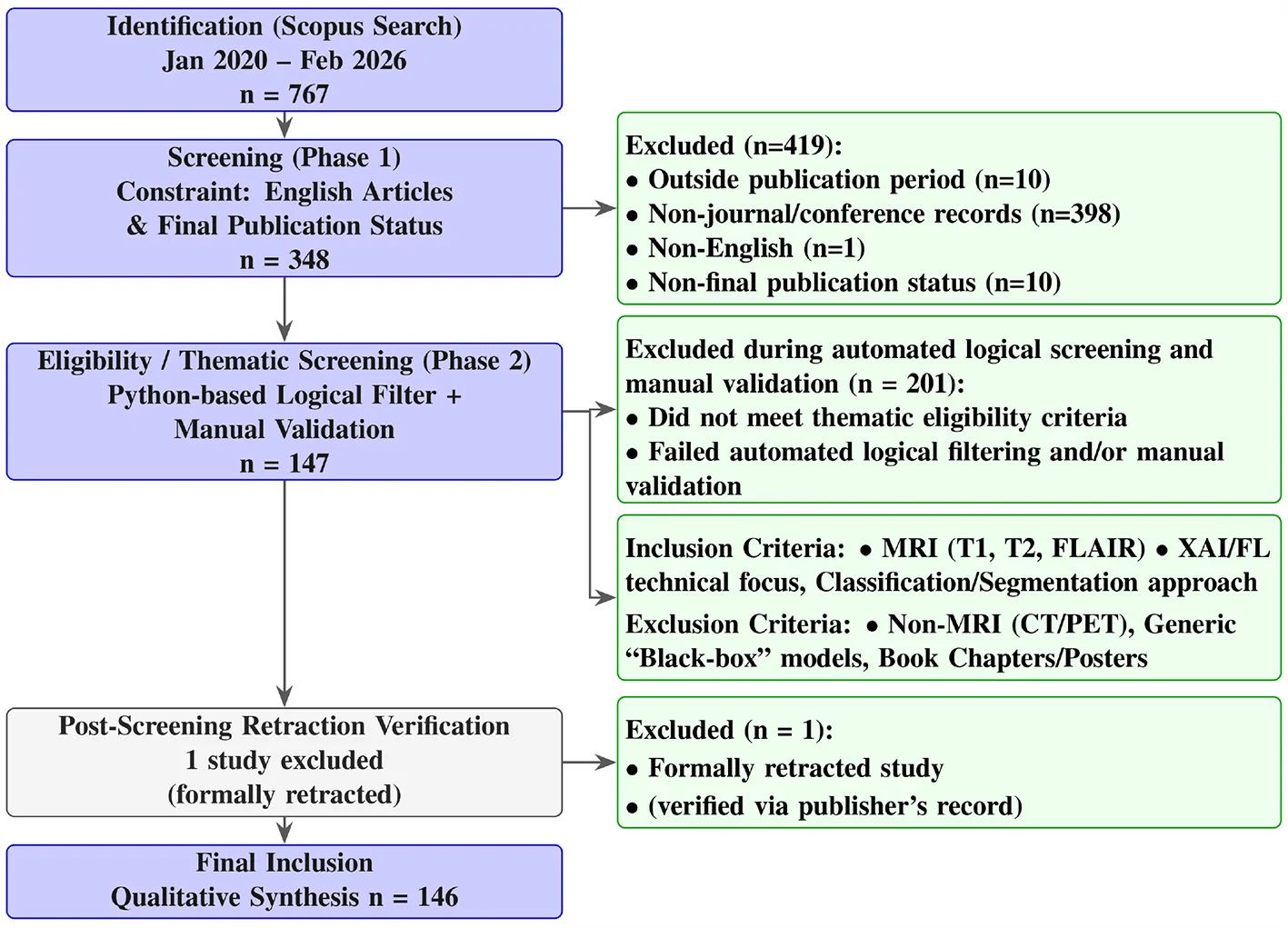
PRISMA-based multi-stage literature selection and screening workflow employed in this systematic review.

Overall workflow for screening and assessing eligibility was conducted following PRISMA-based (Figure 3) guidelines for evidence identification and selection to ensure enhanced methodological transparency and replicability.

After this process of final selection based on quality evaluation, the studies were analyzed for key themes in the research field, trends in architecture evolution, interpretability methods, privacy techniques, and novel methodological gaps in building trustworthy AI systems for brain tumor detection applications. Specifically, emphasis was laid upon how recent works manage to strike a balance between prediction, explainability, multimodality, privacy, and clinical adaptability in brain tumor analysis applications. The thematic analysis indicated a heavy research focus on frameworks driven by explainability and visualizing interpretability techniques, while privacy-preserving and distributed learning paradigms have been relatively neglected. This gap implies an apparent deployment challenge in clinical AI applications where the field of interpretability has grown very fast, but large-scale multi-institutional learning is hindered due to concerns related to privacy. Apart from the discussed architectural and explainability approaches, there was an increasing tendency toward using multi-sequence MRI analysis in order to characterize clinically meaningful tumors. There appears to be a pattern emerging in the literature indicating that multi-sequence architectures usually provide better anatomical context and are interpretable compared to single-sequence architectures. To further consolidate the major thematic trends and unresolved methodological limitations identified across the reviewed literature, Table 4 summarizes the dominant research themes, emerging research directions, and persistent research gaps in trustworthy brain tumor detection using AI.

**Table 4:** Key research themes, emerging trends, and identified research gaps.

Research theme	Ratio	Emerging 2025–2026 trend	Identified research gap
Explainable AI (XAI)	37.7%	Intrinsic attention-based interpretability frameworks	Limited quantitative validation and uncertainty-aware explanation mechanisms
Deep learning frameworks	34.9%	Advanced CNN, hybrid CNN, and transfer-learning frameworks	Limited external validation and clinical generalization
Privacy & Federated learning	13.7%	Differential privacy and decentralized collaborative learning	Performance degradation under strong privacy constraints and limited clinical-scale deployment
Advanced architectures	10.3%	Vision transformers and Mamba-based state-space models	Limited validation on heterogeneous and privacy-sensitive medical datasets
Multi-modal integration	3.4%	Cross-modal attention and volumetric MRI fusion	High computational complexity and scalability challenges in 3D fusion pipelines

An evolution map of the cited literature is illustrated in Figure 4. As can be seen from the timeline, there has been a gradual shift of research on MRI analysis of brain tumors from CNN-driven systems to explainable and privacy-sensitive trustworthy AI systems.

**Figure 4:**
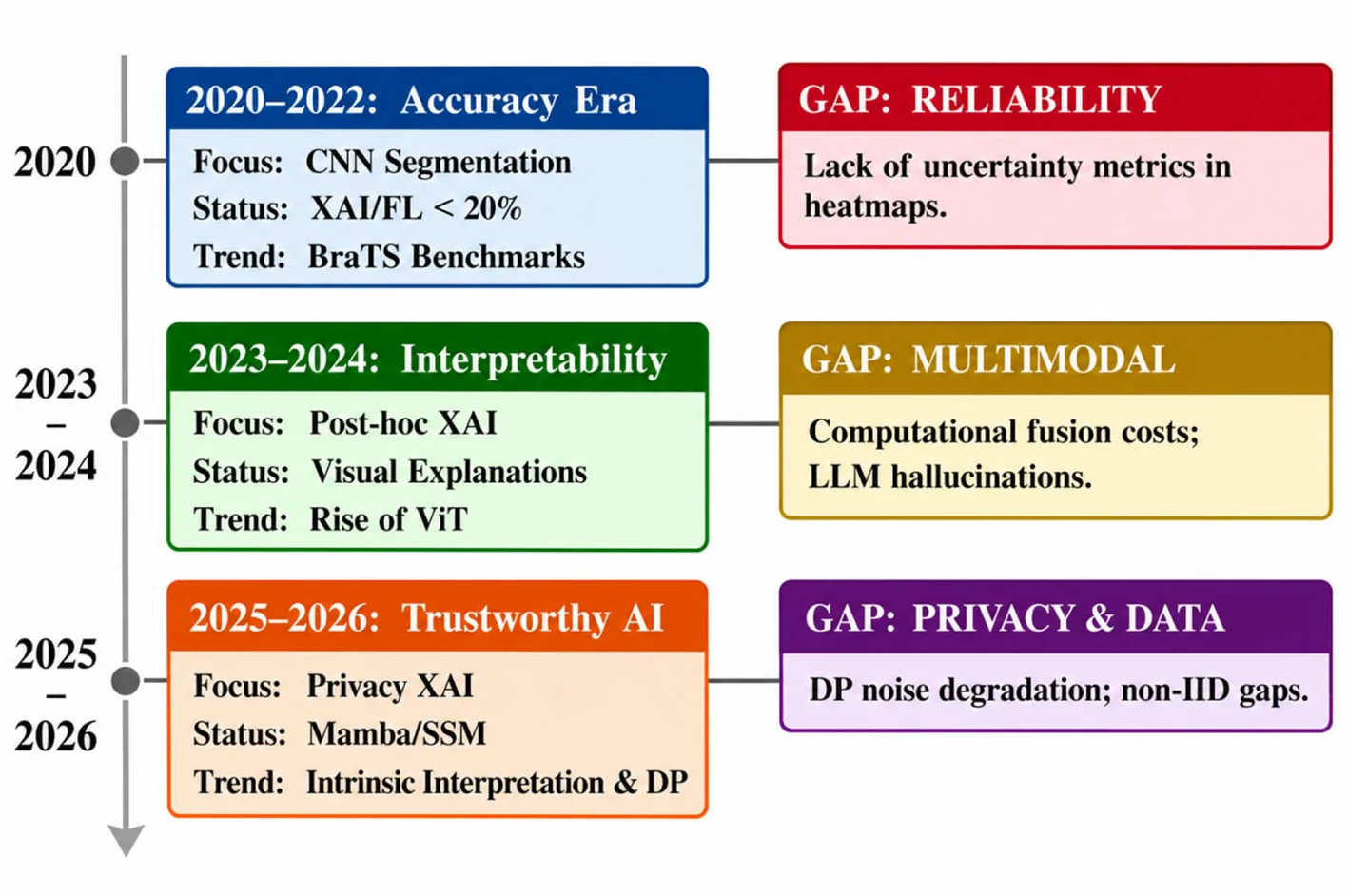
Evolution of brain tumor MRI analysis research from 2020 to 2026, illustrating the transition from CNN-based accuracy-oriented approaches toward explainability-driven and privacy-aware trustworthy AI frameworks.

A rise in the trend of developing clinically interpretable AI models for brain tumor MRIs was also observed during 2024—2026. Specifically, the study has found an increase in the interest in Vision Transformers, state-space models like Mamba, and privacy-sensitive collaborative learning models.

Each included study was assigned to one primary research theme according to its dominant methodological focus. Therefore, the categories in Table 5 are treated as mutually exclusive, and the reported percentages are calculated using the 146 included studies as the denominator and sum to 100%. For quantitative thematic analysis, the denominator was the final set of 146 included studies. Each study was assigned to one primary research theme based on its dominant methodological focus. The five resulting categories were therefore mutually exclusive, with the category counts summing to 146. Percentages were calculated as the number of studies within each category divided by 146 and multiplied by 100.

**Table 5:** Distribution of reviewed studies across core research themes.

Research theme	Number of studies	Percentage (%)
Explainable AI (XAI)	55	37.7
Deep learning frameworks	51	34.9
Privacy and federated learning	20	13.7
Advanced architectures (transformers/Mamba)	15	10.3
Multi-modal integration	5	3.4
Total	146	100.0

The quantitative thematic analysis of the 146 included studies identified Explainable AI (XAI) as the largest research theme, comprising 55 studies (37.7%), followed by deep learning frameworks with 51 studies (34.9%). Privacy and Federated Learning accounted for 20 studies (13.7%), while advanced architectures based on Transformers and Mamba accounted for 15 studies (10.3%). Multi-modal integration was represented in 5 studies (3.4%). Because each included study was assigned to one primary research theme according to its dominant methodological focus, these categories are mutually exclusive and the percentages sum to 100%. For dataset- and task-frequency analyses, the individual study was used as the basic counting unit. Each included study was counted once for each distinct dataset it reported, irrespective of the number of experiments or MRI sequences associated with that dataset. Thus, a study using multiple datasets could contribute to multiple dataset categories, but it contributed at most one count to each specific dataset category. Consequently, dataset frequencies were not mutually exclusive and were not expected to sum to the total number of included studies. For task-frequency analyses, studies addressing multiple tasks were counted in each applicable task category; therefore, task-specific frequencies and denominators were also not mutually exclusive. These counting conventions were applied consistently to the dataset distribution and dataset–task analyses.

A study-level evidence matrix covering all 146 included studies is provided as Supplementary File. The matrix records publication details, study year, tumor type, dataset used, task, architecture, XAI method and evaluation, privacy type, validation strategy, external validation status, reported metrics, major limitations, and the assigned research classification.

## 4 Results and analysis

The final set of studies consisting of 146 papers was systematically evaluated to determine prevailing trends in architectural designs, methodological improvements, validation techniques, and open areas of investigation within the realm of brain tumor MRI analysis. Based on the results, there is an increasing diversification beyond conventional CNN-based approaches toward Transformers, multimodal architectures, explainable AI, and privacy-preserving learning.

### 4.1 Deep learning-based brain tumor analysis

The field of deep learning has revolutionized the field of brain tumor analyses through improved automatic methods for segmentation, classification, prognosis, and treatment planning based on imaging data obtained from neuroimages. Recent developments in deep learning for brain tumors increasingly incorporate approaches beyond conventional CNN-based classification, including explainability, multimodal learning, optimization techniques, and clinically adaptive AI paradigms. In order to appreciate the evolution of these different paradigms, the studies reviewed herein are classified under various headings.

#### 4.1.1 CNN and transfer learning-based frameworks

Early researches in brain tumor detection through deep learning techniques mainly relied on the CNN architecture and transfer learning methods for detecting tumors, classifying multiple classes, and segmenting tumors. Pretrained backbone networks, including ResNet, DenseNet, EfficientNet, and YOLO, were used for better extraction and classification capabilities of limited MRI data. Research works such as (Saifullah et al., 2025; Awasthi et al., 2025; Zhang et al., 2026; Sumona et al., 2025; Disci et al., 2025; Ferreira et al., 2025; Ishaq et al., 2025; Mujahid et al., 2024; Chourib, 2025; Nobel et al., 2025) provide evidence of rapid developments in CNN and transfer learning approaches toward lightweight hybrid models with high explainability for brain tumors classification, detection, and segmentation. Most researchers used the pre-trained models like ResNet50, EfficientNet, InceptionResNetV2, Xception, MobileNetV3, and variants of YOLO networks to optimize feature extraction while lowering the training difficulty owing to limited MRI data sets. Transformer-assisted YOLO frameworks along with RDXNet and CancerNet architectures extended traditional CNN pipelines by combining the attention mechanism and transformer layers for multi-branch feature fusion. Likewise, transfer learning approaches and optimizations like recursive feature elimination, stacked autoencoder, and morphological operations were extensively used to enhance classification separability, reduce computational cost, and improve the explainability of the models using Grad-CAM, SHAP, and saliency mapping. Overall, these works show that CNN and transfer learning approaches continue to dominate research in brain tumor detection using AI owing to their high efficiency and performance in multiclass classification tasks.

#### 4.1.2 Explainable and interpretable deep learning models

In order to boost the level of trust in clinicians and enhance decision-making, the latest research has adopted an approach involving the incorporation of XAI methods like SHAP, LIME, Grad-CAM, CAM, and neuro-fuzzy reasoning in deep learning workflows. The goal was to detect biologically relevant tumor areas and generate explainable interpretations along with predictions. These studies, (Saeed et al., 2023; Hardani et al., 2024; Zheng et al., 2023a; Valerio et al., 2025; Šefč́ık and Benesova, 2023; Gaur et al., 2022; Mayeta-Revilla et al., 2025; Mazhar et al., 2026; Jahan et al., 2025; Gao et al., 2025) together show that there is an increasing trend toward moving away from accuracy-driven deep learning models for the application of brain tumor detection using AI toward explainable and biological models. Many of these papers adopted techniques like Grad-CAM, SHAP, LIME, CAM, neuro-fuzzy reasoning, or explanation-guided learning methods to ensure better explainability and provide better insight into tumor-related areas of interest. Although the MGMT methylation paper cast serious doubt on the explainability techniques used today due to the poor correlation of model attention with biologically interesting tumor characteristics, frameworks like CSF-Glioma and explanation-guided learning tried to achieve explainability during the model learning process itself rather than relying on *post-hoc* techniques. At the same time, segmentation-to-explanation models extended explainability further by integrating large language models for human-comprehensible reporting, while histopathology-based frameworks like PGE-Net and diffuse glioma classifier models took pathological cues and multi-scale explainability techniques to enhance explainability in WSIs. Nevertheless, despite significant developments in the implementation of Explainable AI in these models, several major issues remain outstanding within the frameworks. For example, most of the methods for generating explanations are *post-hoc* visualizations that are not rigorously validated from a biological or clinician-oriented perspective, meaning that it is not always clear whether the highlighted portions correspond to causative features or accidental imaging patterns. Additionally, combining various explainability tools, such as SHAP, LIME, Grad-CAM, and fuzzy inference systems, results in considerable computational overhead as well as potential disagreements between different explainers. Although some of the studies report exceptionally high classification accuracy rates, most of the models are still highly dependent on specific curated datasets like BraTS, DeepHisto, and Kaggle MRI, thus reducing their ability to operate under realistic clinical conditions and variations in image acquisition or staining procedures. In turn, the application of language learning model-generated explanations poses risks associated with potential hallucination, prompt dependency, and factual inaccuracies, even with anti-hallucination measures applied. Meanwhile, histological explainability approaches are vulnerable to staining and patch-related errors, whereas MRI explainability approaches have to overcome challenges related to noisy imaging procedures and heterogeneous tumors. As seen from Figure 5, current explainable AI frameworks for brain tumor detection use multimodal MRI, histopathological data, genomics, *post-hoc* visualization techniques, inherent explanation capabilities, and approaches to multi-XAI integration. In addition to that, Figure 5 points out the main problems of such systems, namely explainers' discrepancies, hallucinations, and mismatch between visual explanations and biological reasons of tumorous changes.

**Figure 5:**
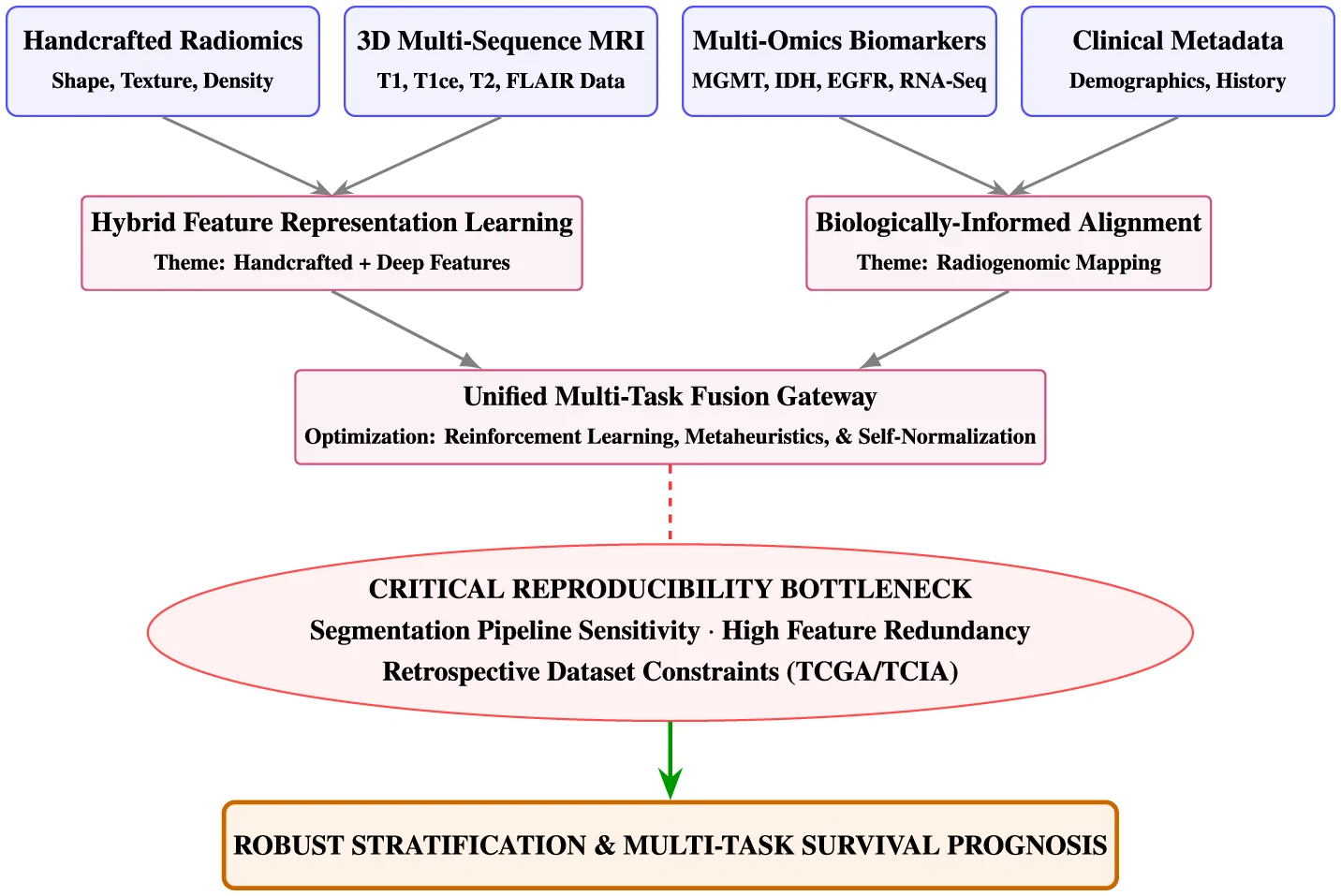
Overview of Explainable Deep Learning Systems for Brain Tumor Analysis, showing how multimodal input data is incorporated into *post-hoc* explainability techniques, intrinsically interpretable models, and LLM-based report generation systems. The figure also emphasizes the limitations in terms of explainer disparity, hallucination risks, and the mismatch between visual attention maps and biological causal mechanisms.

Despite the increased explainability in these models, researchers found out that using images alone might not be enough to describe the heterogeneity of tumors.

#### 4.1.3 Hybrid, radiomics, and multimodal learning frameworks

The hybrid and multimodal models utilized imaging attributes together with radiomics, genomics biomarkers, clinical metadata, and multi-omics data to enhance glioma grading, molecular stratification, and survival prognosis. Such methods capitalized on different feature descriptions to better reflect the biological heterogeneity of brain cancer. The findings from studies such as (Renugadevi et al., 2025a; Zeng et al., 2024; Yin et al., 2025; Wang et al., 2025; Wan et al., 2025; Bilalov et al., 2025; Fan et al., 2025; Onciul et al., 2025) show an emerging trend toward multimodal brain tumor detection methods that combine radiomics, deep learning algorithms, clinical metadata, genomic biomarkers, and multi-omics. Most of the works have shown that the combination of handcrafted radiomics with CNN-based deep features provides better survival predictions than using only one of these methodologies individually. Multimodal fusion methods, on the other hand, that combine radiomics with MRI sequences, morphological characteristics of the tumors, genomics (MGMT, EGFR, IDH), transcriptomics, and single-cell sequencing were also more effective in representing the complexity of gliomas, treatment responses, and survival. Other works such as GlioSurvQNet and multi-omics survival methods have introduced reinforcement learning, metaheuristic learning, and self-normalizing methods to improve feature robustness and survival prediction. The use of multitasking techniques was shown to be capable of reducing redundancy by utilizing correlation among the related clinical outcomes. However, in spite of their improved ability to predict prognosis, these hybrid approaches are still burdened by several critical issues. For instance, the inclusion of thousands of radiomic, genomic, and multi-omics features has increased the need for computational power, feature redundancy, and standardization of preprocessing pipelines. While some studies benefited from multi-center validation in improving their robustness, most of the systems heavily rely on retrospective public datasets like TCIA and TCGA, limiting their generalizability across different imaging environments and techniques. Also, while some frameworks were able to overcome the problem of dependence on good segmentation quality, some of them exhibited high sensitivity to this parameter, especially those utilizing radiomic features obtained via automatic segmentation pipelines, highlighting another critical issue of reproducibility of survival analysis based on hybrid systems. While interpretability was enhanced using SHAP values, multitask learning, and other methods of feature interpretation, several frameworks were only semi-transparent and did not incorporate any biologically validated mechanisms for explaining the predictions of the models. Lastly, the addition of genomics and transcriptomic data to radiomic predictions could present challenges for the infrastructure of such systems, making them difficult to deploy in practice. Figure 6 highlights the integration of radiomics, multimodal MRI, genomic markers, and clinical information into hybrid fusion models in the context of glioma grading, molecular characterization, and survival analysis. Moreover, it brings to attention important reproducibility issues such as feature redundancy, segmentation sensitivity, and reliance on retrospective datasets.

**Figure 6:**
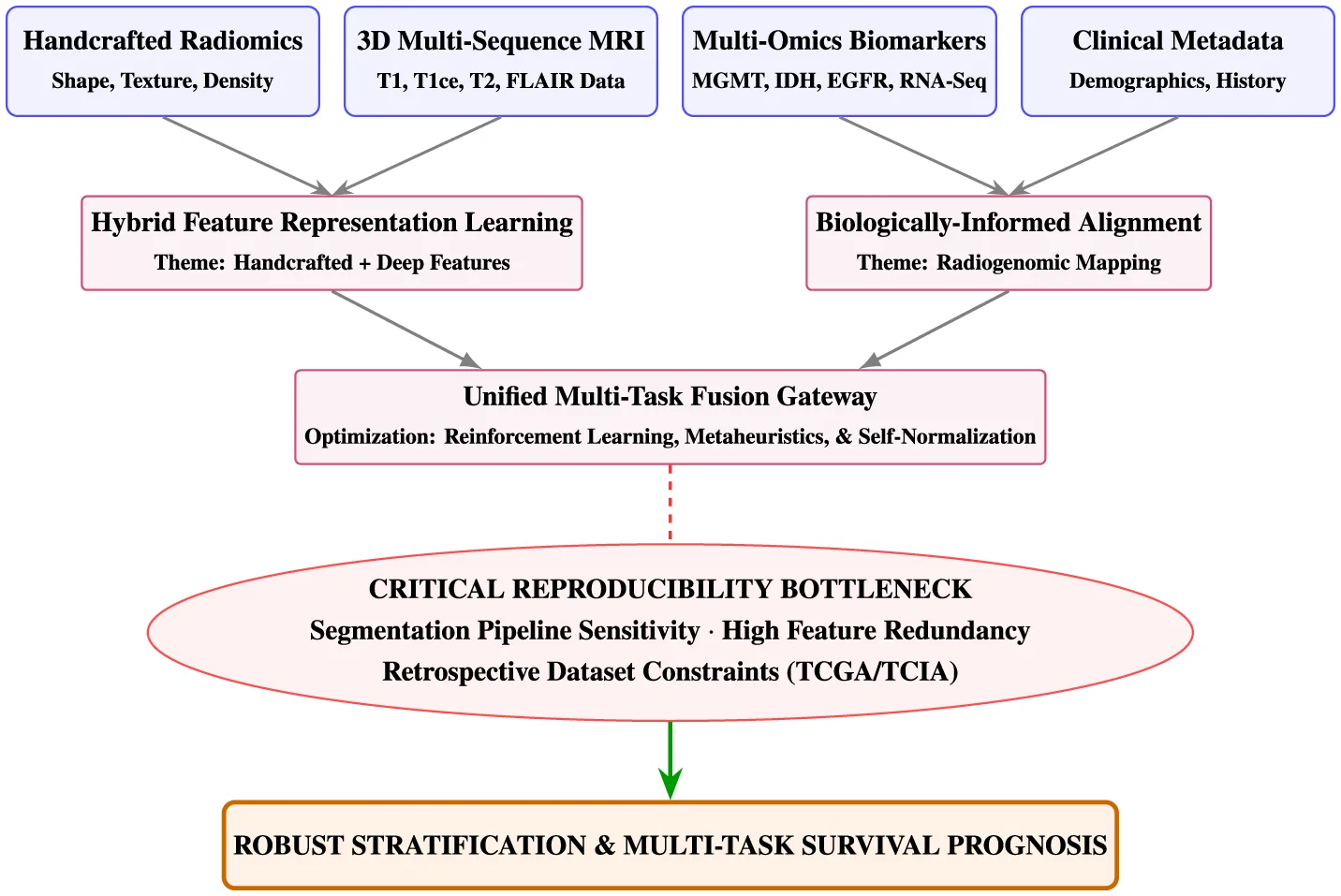
Hybrid and multimodal AI approaches in brain tumor detection using radiomics, multimodal MRI analysis, genomic information, and metadata to perform glioma grading, biomarker discovery, and prognostic analysis. Limitations in the use of such models include sensitivity to image segmentation, redundant feature engineering, and dependence on retrospective datasets.

However, due to some constraints regarding the availability of training samples, domain adaptation, and partial MRI scans, there was a need for more flexible and resilient learning strategies.

#### 4.1.4 Advanced learning paradigms and robust AI systems

Recently, approaches such as self-supervised learning, semi-supervised learning, few-shot learning, contrastive learning, uncertainty estimation, and foundation models have been developed to boost robustness in scenarios with minimal annotations and diverse clinical settings. This was meant to advance the domain generalization and robustness capabilities of the neuroimaging workflow. Researches like (Renugadevi et al., 2025c,b; Tak et al., 2024; Vaidya et al., 2025; Tupe-Waghmare et al., 2021; Wu et al., 2025; Hu et al., 2023; Nag et al., 2024; Chrysochoou et al., 2026; Park et al., 2024; Chen et al., 2024) collectively illustrate the evolving development of AI models designed to address challenges such as limited annotations, domain shifts, uncertainty quantification, and incomplete MRI scans in brain tumor detection. Almost all research papers departed from traditional CNN training methodologies to include concepts of self-supervised learning, few-shot learning, contrastive learning, reinforcement learning, adversarial network-based data augmentation, uncertainty-aware model calibration, and foundation model pre-training to increase the feature generalization capability of AI models. Models like MAML, SimCLR-Twin Critic DDPG, and semi-supervised multitasking learning emphasized the need for data-efficient learning under limited labeled data. Whereas, MCN and deep ensemble learning methods focused on addressing one of the most challenging issues in clinical settings—robustness under distribution shifting and out-of-distribution samples. Likewise, foundation models and missing MRI data researches showed how self-supervised pre-training and modality robust learning could significantly increase the scalability and adaptability of AI models in heterogeneous imaging scenarios. Nonetheless, there remain several significant drawbacks associated with even these state-of-the-art learning methodologies. In particular, current techniques generally incorporate highly complex architectures based on multi-step pipelines including segmentation ensembles, contrastive pretraining, GAN data augmentation, transformers, reinforcement learning, and uncertainty calibration, thus hindering reproducibility and increasing computational burden. On the other hand, while self-supervised and few-shot learning have been shown to enhance generalization under limited sample sizes, recent studies still reported performance deterioration under extreme domain shifts, noise, and heterogeneous clinical protocols. GAN augmentation and generative MRI synthesis methods pose additional risk for potential artifacts and clinically deceptive results. Though uncertainty aware ensembles and calibration systems helped increase estimation reliability, their practical implementation might prove difficult in resource-limited medical settings due to greater inference demands. Finally, although some external validation efforts have been made, the reliance on benchmarking databases such as BraTS and TCIA remains prevalent, compromising translational accuracy in practice. As seen from Figure 7, modern, robust AI approaches are using more sophisticated concepts like few-shot learning, semi-supervised learning, contrastive learning, uncertainty quantification, data generation techniques, and pretraining on foundation models. Additionally, some operational concerns are presented in Figure 7—for example, computational complexity during inference, generative artifacts, and reliance on benchmarks.

**Figure 7:**
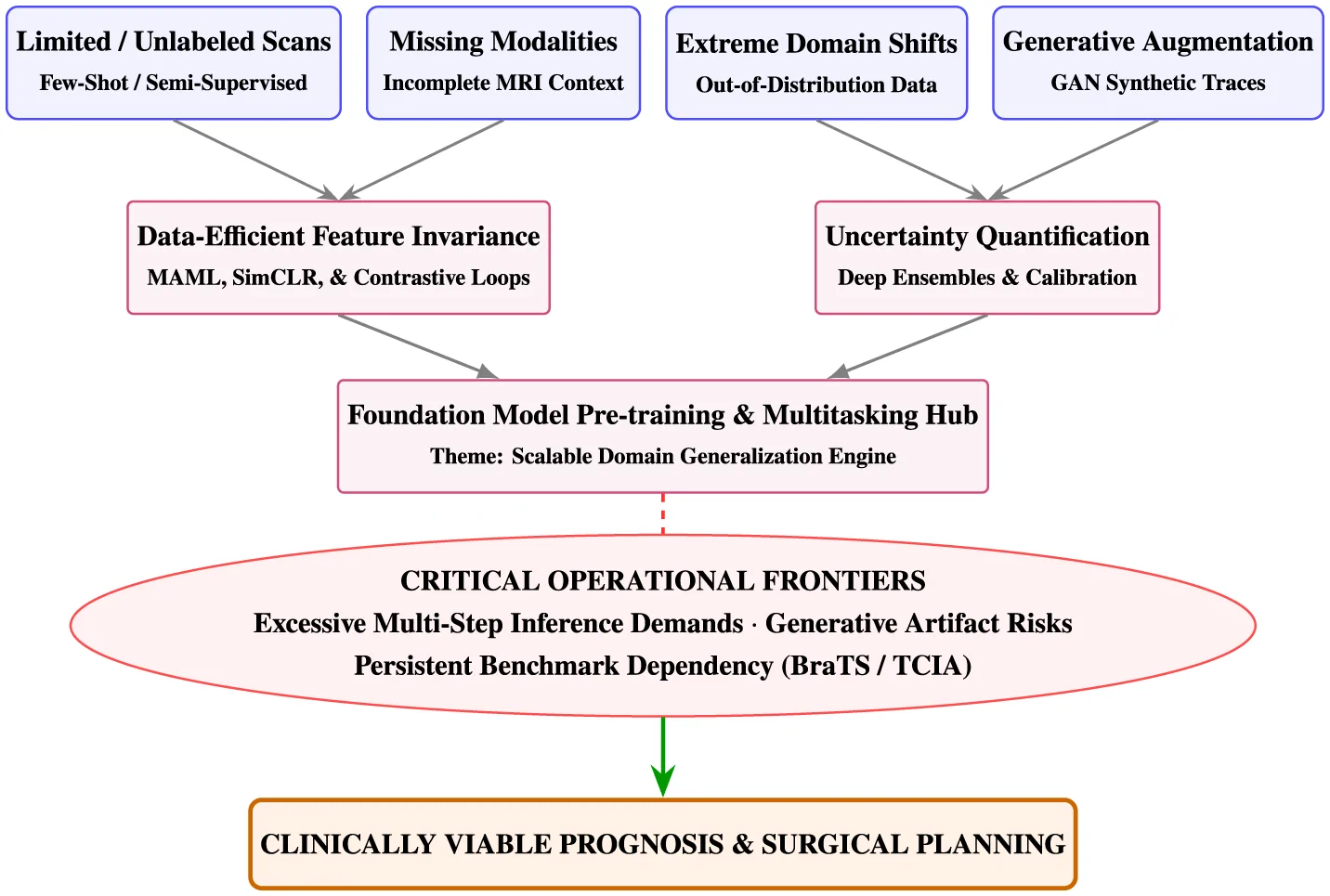
Conceptual depiction of advanced robust artificial intelligence frameworks incorporating few-shot learning, semi-supervised learning, contrastive learning, uncertainty modeling, data augmentation, and foundation model pretraining for achieving better domain and modality generalizability.

In conclusion, all of these works indicate that future trustworthy AI-based brain tumor detection solutions should be oriented toward scalable generalization, computational efficiency, clinically grounded uncertainty estimation, and multi-institutional evaluation. As the robustness levels increased, the focus of research began to move toward creating clinically viable methods for prognosis prediction and surgery planning.

#### 4.1.5 Clinical translation, prognostic modeling, and workflow-oriented systems

AI-oriented clinical workflow studies included efforts toward integrating AI in the prediction process, pre-processing procedures, surgery planning, and treatment decision-making support. Such systems were designed to be practical, robust in pre-processing, automated in workflows, and effective in providing decision support for brain tumor detection practices. The combination of studies including (Thakur et al., 2020; Acquarelli et al., 2022; Herr et al., 2024; Rajesh et al., 2026; Mut et al., 2024; Zhu et al., 2022; Pourmadadi et al., 2025; Sagiroglu et al., 2025) for example, highlights the trend toward the advancement of translational workflow-based AI in brain tumor detection. The studies in particular which were aimed at developing AI solutions with better robustness for deployment in clinical workflows included modality-agnostic preprocessing, segmentation and diagnosis for multiple tasks, workflow automation, and aiding prediction of surgical outcomes and treatment response. For instance, the study which investigated the craniopharyngioma invasiveness framework, along with the study about glioblastoma surgery decision making showed the gradual adoption of AI to aid pre-operative planning and clinical decision making support. Furthermore, the study which used MRSI CNN-based model along with the digital pathology length scale analysis showed an effort toward feature learning that is biologically meaningful rather than performance oriented. Lastly, the review of AI-driven nanocarriers for theranostic and targeted drug delivery further showed the application of AI in brain tumor detection outside of image processing for predicting biological processes and disease. Nevertheless, in spite of the progress in terms of translation-oriented approaches, there is also a number of crucial limitations in regard to such systems. First, many workflow-oriented frameworks appear to be extremely dependent on retrospective cohorts of patients and controlled imaging settings within medical institutions. Moreover, while the introduction of multimodal decision-making models and clinically oriented imaging significantly enhances the level of decision support, it introduces a lot of unnecessary complexity. Indeed, as the case of surgery-oriented review has shown, current brain tumor detection systems cannot perform similar complex reasoning in relation to patients' conditions and needs. At the same time, although modality-independent preprocessing and explainable imaging models help increase adaptability of AI systems to various challenges, the models of segmentation and prognosis still show high vulnerability in regard to certain imaging artifacts and scanner differences. As for theranostic and multiomics-integrated approaches, there are no doubts about their future success; however, the question of regulatory approval and scalability is yet to be resolved. Figure 8 highlights how recent paradigms have begun to incorporate multimodal imaging, modality agnostic preprocessing, digital pathology, and theranostics into one pipeline-oriented approach to aid prognosis, surgery planning, and clinical decision-making. Figure 8 further indicates that while the recent advancements have enabled increased automation and biologically relevant learning within these approaches, important bottlenecks that include dependence on retrospective cohorts, vulnerability to imaging artifacts, scalability issues, and regulatory constraints persist.

**Figure 8:**
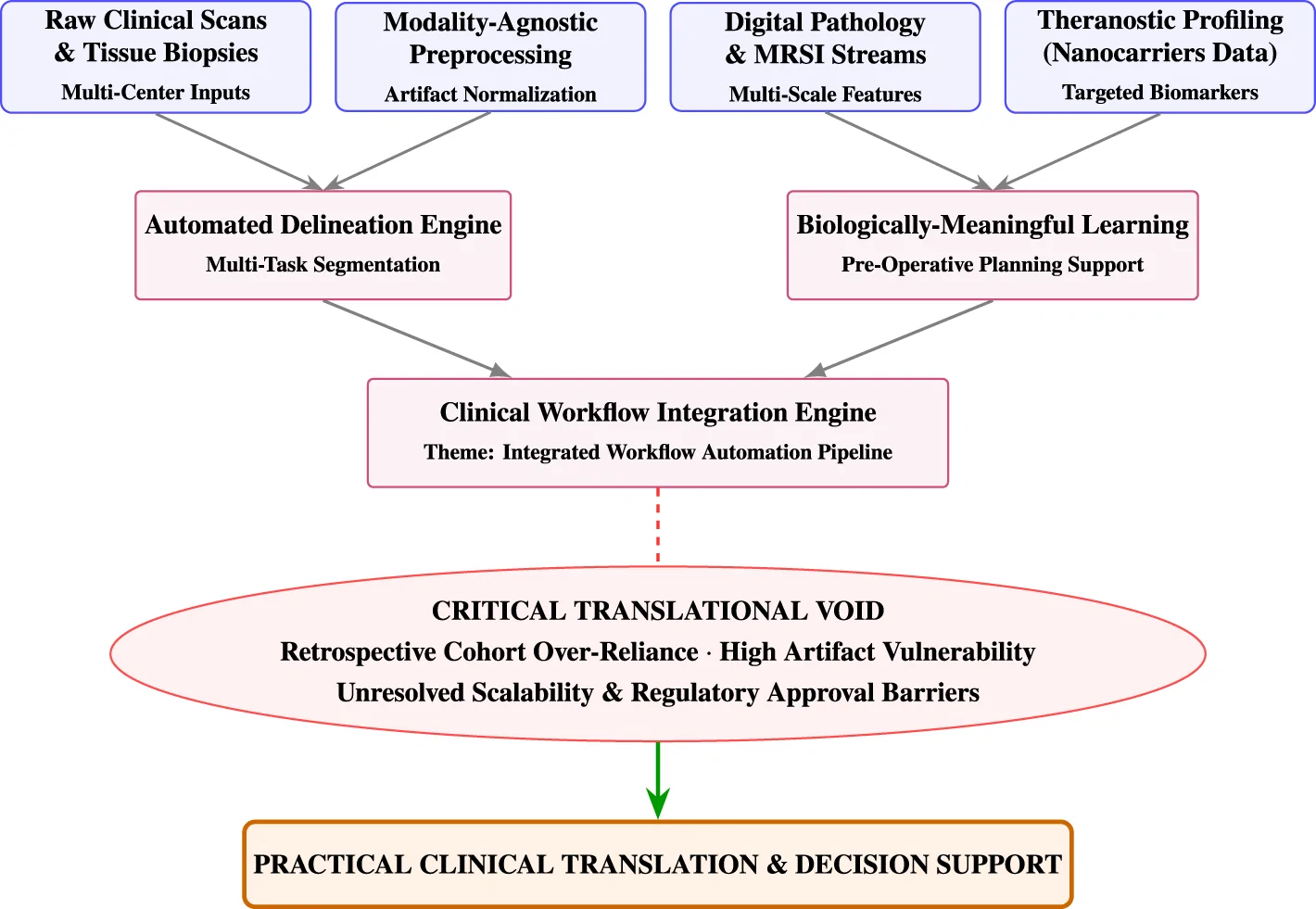
Translational Workflow of brain tumor detection using AI Frameworks that incorporate multimodal imaging, robust pre-processing, digital pathology, and therapeutic-diagnostic profiling for prediction of prognosis, surgery planning, and clinical decision-making.

In order to achieve greater accuracy in prediction and greater computational efficiency, current research in brain tumor detection using AI systems began to adopt optimization techniques and hybrid intelligence approaches.

#### 4.1.6 Hybrid and optimization-driven brain tumor detection

The brain tumor detection approaches based on optimization were combined with swarm intelligence, reinforcement learning, feature selection, metric learning, and hybrid deep learning methods in order to strengthen classification and prognosis capabilities. These techniques were developed to increase feature separability, avoid redundancy, and provide adaptability when dealing with diverse brain tumors. Works like (Wang et al., 2024; Li et al., 2025; Yonar, 2025) provide additional evidence for an increasing trend toward designing hybrid and optimization-based brain tumor detection using AI solutions which incorporate transfer learning, machine learning classifiers, survival models, and feature optimization techniques. The study with hybrid multiclassifier architecture proved the effectiveness of using a combination of super-resolution reconstruction, transfer learning, and machine learning classifiers, such as LightGBM, compared to traditional CNNs in terms of improving classifier robustness and automation. Meanwhile, DeepSurv prognostic model confirmed that neural network-based survival modeling approaches are superior to traditional Cox regression and random survival forest when applied to diffuse gliomas dataset with missing clinical data. On the other hand, DenseWolf-K model showed how discriminative feature learning techniques based on swarm intelligence optimization and metric learning can enhance classifier separability while minimizing redundancy. All mentioned works prove that the contemporary research in the area of brain tumor detection using AI, tends to develop hybrid solutions combining deep learning with optimization and predictive techniques. Despite their enhanced ability to predict, these paradigms still have to overcome a number of major issues. The models still rely significantly on retrospective databases and MRI benchmarks, thus posing risks associated with generalization in terms of imaging variability. Moreover, the use of numerous optimization routines, transfer learning frameworks, and feature selection techniques results in elevated architectural and computing costs, possibly compromising reproducibility among healthcare facilities. While methods of model explainability, including occlusion sensitivity maps and visualization, contributed greatly to the transparency of hybrid paradigms, in the vast majority of cases, the obtained explanations were still *post-hoc* and lacked biological validation. Finally, although hybrid optimizations enhanced the classification performance, their extremely high efficiencies could, to some extent, be attributed to the presence of fine-tuning on specific datasets and experimentally constrained conditions, which might affect their clinical applicability.

### 4.2 The evolution of architectural backbones

The recent architectures in deep learning are increasingly utilizing attention mechanisms, transformers, and multi-branch 3D networks to enhance spatial features and understand the complexities of brain tumors. Table 6 summarizes architectural capabilities and methodological characteristics using the predefined qualitative coding framework described in Section 4. The symbols ✓, △, and × denote strong/full support, partial or limited support, and absent, weak, or insufficient evidence, respectively. It also emphasizes the recent shift toward the use of hybrid and transformer-based architectures for brain tumor analysis. Although there have been great strides in architecture innovations, most of the studies considered lack proper external validation, poor generalization assessment, and inadequate explainability inclusion. This shows that architecture advancements are outpacing clinical reliability and practicality considerations. Although transformer architectures provide substantial improvements in contextual feature representation, their computational intensity and memory constraints may hinder their practical application in resource-limited clinical settings.

**Table 6:** Architectural innovation and validation rigor in emergent literature.

Core architecture	**Spatial comp**.	**Multimodal integration**	Task scope	XAI support	Validation rigor	Data generalization	Computational burden	Clinical readiness
Geometric DL [Mod-SE (2)] (Angelina et al., 2026)	✓	×	Classify + Segment	×	×	×	△	×
Self-Attn. MoE (Liao et al., 2026)	△	✓	Radio-genomic	△	×	△	△	×
3D Attention U-Net (Alwadee et al., 2025)	✓	✓	Segment	△	×	×	✓	△
ResNet + spatial/channel attention + transformer context (Majeed et al., 2025)	△	×	Classify	△	×	×	△	×
Vision Transformer (ViT) for SRS response prediction (Volovăţ et al., 2025)	△	×	Prediction	×	×	×	△	×
Gabor-enhanced U-Net with dual attention (Chamseddine et al., 2025)	✓	✓	Segment	×	×	×	△	×
Hybrid CNN + Transformer (DeiT-Tiny) (Gutta and Yathirajam, 2026)	△	✓	Classify	△	×	×	△	×
Graph Attention Network (BioGAT-LGG, GATv2) for multi-omics integration (Hamid et al., 2026)	△	✓	Classify	△	△	×	△	×
Edge-Aware Transformer (EA-Trans) with adaptive self-attention (Abbasi et al., 2026)	✓	×	Classify	△	△	△	△	×
Multi-Modal 3D Attention Network (MM-3DAttNet) (Ramasamy et al., 2026)	✓	✓	Prediction	△	△	×	△	×
Hybrid CNN + vision transformer with XAI module (Aiya et al., 2025)	✓	✓	Segment	✓	×	×	△	△
Swin Transformer + V-Net Hybrid (SwinVNETR) (Maria Nancy and Sathyarajasekaran, 2024)	✓	✓	Segment	×	×	×	△	×
Hybrid 3D CNN–Transformer (3D U-Net + V-Net fusion with transformer blocks) (Sharma and Ameta, 2025)	✓	✓	Segment	×	×	×	△	×
Hybrid ViT–CapsNet framework (capsule-transformer integration) (Simran et al., 2025)	✓	✓	Classify	×	×	×	△	×
Fine-Tuned Vision Transformer (FT-ViT) with transfer learning (Priya and Vasudevan, 2024)	△	×	Classify	×	×	×	△	×

A series of research works highlights architectural innovations for image-based tumor characterization (Alwadee et al., 2025; Majeed et al., 2025; Chamseddine et al., 2025). To exemplify, Abbasi et al. (2026) introduce an Edge-Aware Transformer, which leverages the benefits of edge-sensitive tokenization and wavelet-based convolutional operations for more effective tumor boundary detection, addressing the drawbacks of CNNs for handling the irregular of tumors. Analogously, the research work covered under the Mod-SE(2)-based framework (Angelina et al., 2026) is centered around the improvement of the accuracy of the segmentation results using geometric deep learning with the aid of attention mechanisms, underscoring the challenges associated with the accuracy of the boundaries and the computational costs of the 3D tumor segmentation task. On the other hand, the research work conducted by Gutta and Yathirajam (Gutta and Yathirajam, 2026) analyses the application of hybrid CNN-Transformer-based architectures for glioma grade-level classification, underscoring the challenges associated with the accuracy of the results, though the work is based on a single dataset, namely, the BraTS dataset. Studies by Liao et al. (2026) and Volovăţ et al. (2025) further explored transformer-based representation learning for tumor classification and prediction tasks.

Beyond architectural innovations in image-based analysis, recent studies increasingly emphasize multi-modal and multi-omics integration to capture the biological heterogeneity of brain tumors. Hamid et al. (2026) propose a correlation-based Graph Attention Network (BioGAT-LGG) that incorporates various omics information to classify the subtypes of lower-grade gliomas. This addresses the biological heterogeneity that imaging-based models may not be able to capture. However, the model's dependence on limited data sets and the interpretability of the attention mechanism also point to the difficulties that arise in biological validation. Ramasamy et al. (2026) propose the MM-3DAttNet model, which is a multi-modal 3D attention network that incorporates various MRI sequences such as T1, T1ce, T2, and FLAIR to predict the methylation status of the MGMT gene. The weak supervision at the patient-label level and the lack of uncertainty calibration also present difficulties.

Recent developments in transformer-based and hybrid volumetric architectures further demonstrate the shift toward more context-aware learning frameworks for brain tumor analysis. There is a consensus among Maria Nancy and Sathyarajasekaran (2024) and Sharma and Ameta (2025) that traditional Fully Convolutional Neural Networks (FCNNs) are not sufficient for the purpose of capturing the dependencies in volumetric MRI data. To address the issue, researchers are shifting toward hierarchical Swin Transformers and 3D U-Net/V-Net hybrid models. However, the continued reliance on skip connections reflects architectural dependencies inherited from earlier segmentation frameworks. One of the key points that have been observed from the works of Maria Nancy and Sathyarajasekaran (2024) and Sharma and Ameta (2025), and the previous works such as Capsule-TransUNet (2025) is the over-reliance on BraTS datasets (2019-2021). While the Capsule-TransUNet achieves a near-perfect accuracy of 99.8%, the absence of external validation raises concerns regarding potential overfitting and limited real-world generalizability. Although Aiya et al. (2025) proposes *post-hoc* explanation tools like saliency maps to establish a form of “clinical trust,” there appears to be a disconnect between model complexity and interpretability. As model architectures continue toward Fine-tuned Vision Transformers, as discussed by Priya and Vasudevan (2024), patch-level processing becomes more difficult to provide biological explanations. As Simran et al. (2025) also discussed, despite their ability to perform well on standard tasks, these advanced architectures still face issues with clinical reliability for diagnosing uncommon tumor types. In summary, the discussed literature reveals a definite shift toward transformer-based and multimodal models; yet, the issues surrounding external validation, robustness to explainability, uncertainty estimation, and practicality hinder the application of such novel brain tumor detection using AI models in the clinical realm.

### 4.3 Bridging the trust gap: Explainable AI (XAI) in brain tumor detection

Approaches such as the combination of convolutional neural networks and explanation methods like Grad-CAM, SHAP, LIME, and attention visualization aim to highlight tumor-relevant areas and help clinicians better understand the decisions made by the models (Sánchez-Moreno et al., 2025; Pongphaw and Buaphan, 2026; Srinivas and Parvathi, 2025; Shaheema et al., 2025; Samara and Harry, 2025), Saleem et al. (2021), and Safarpour et al. (2026). Though these approaches improve the interpretability of the diagnostic results, many of these approaches are still based on saliency map-based methods, which are not always reliable. Other approaches focus on the development of hybrid approaches for feature engineering and explanation methods, where machine learning classifiers are combined with feature selection methods and explanation methods like Boruta, LASSO, and SHAP to improve the selection of clinically meaningful radiomic features (Rohan et al., 2025; Hosny et al., 2025; Rahman et al., 2024; Song et al., 2022; Hosny and Mohammed, 2025; Mushgil et al., 2025; Aksoy et al., 2025). Though these approaches improve the interpretability of the diagnostic results, they are still based on carefully curated data, which may not be applicable to all situations. Recent approaches have been made to improve the interpretability of the diagnostic results by developing explanation methods for segmentation and multi-task approaches, where tumor localization, segmentation, and classification are combined to improve the interpretability of the results for better tumor characterization in MRI scans (Andrianos et al., 2025; Ali et al., 2025; Cao et al., 2023; Dasanayaka et al., 2022; Ayaz et al., 2024; Bhaskaran and Datta, 2025). These methods have shown better interpretability of the diagnosis but often come with the cost of higher computational complexity and large amounts of annotated data like BraTS. Another direction that is currently being explored is the fusion of explainable AI with more powerful deep learning architectures like ensemble methods, transformer networks, and multimodal methods for more reliable predictions (Gagliardi et al., 2025; Alyatimi et al., 2025; Akgündoğdu and Çelikbaş, 2025; Eder et al., 2022; Gharaibeh, 2025; Gundogan, 2025; Bhuiyan et al., 2025; Haque et al., 2024; Abdulateef, 2025; Charaabi et al., 2025; Haque et al., 2025; Christodoulou et al., 2026; Zhao et al., 2025). Both Desai and Mushrif (2025) and Mahesh et al. (2024) employ CNN architectures based on EfficientNet, using Grad-CAM for improved explainability while classifying brain tumors on MRIs, but whereas Desai and Mushrif (2025) concentrates on comparing various pretrained transfer learning models and optimization methods, Mahesh et al. (2024) highlights its clinical interpretability and reliability through an explainable AI model based on EfficientNetB0. Although these methods have shown better performance and more context with respect to decision-making, there is often a challenge with respect to model complexity and interpretability. Lastly, there is another set of works that focuses on trust-aware AI systems that consider fairness, privacy, and transparency in medical imaging tasks. Although all of these works try to bridge the gap between high-performance AI and its acceptance in the clinic, there is often a lack of standardized evaluation metrics for explainability and clinical validation. Overall, the literature demonstrates substantial progress in integrating XAI with deep learning for brain tumor diagnosis; however, persistent challenges remain in explanation reliability, dataset dependency, computational burden, and the need for clinically interpretable evaluation protocols.

The comparative analysis of the strengths, limitations, and gaps identified in the reviewed XAI papers is summarized below in Table 7.

**Table 7:** Critical analysis and comparison of XAI literature.

References	XAI	Clinical interpretability	Tumor complexity	Computational efficiency	Robust validation	Multimodal integration
Palkar et al. (2024)	✓	✓	✗	✗	✗	✗
Osadebey et al. (2023)	✓	✓	✗	✗	✗	✗
Pan et al. (2022)	✓	✓	✓	✗	✗	✓
Natekar et al. (2020)	✓	✗	✓	✗	✗	✗
Mzoughi et al. (2025)	✓	✓	✓	✗	✗	✗
Li et al. (2024)	✓	✓	△	△	△	×
Lee et al. (2025)	✓	✓	✓	✗	✓	✓
Kina (2025)	✓	✓	✓	✓	✗	✗
Khushubu et al. (2025)	✓	✓	✓	✓	✗	✗
Innani et al. (2025)	✓	✓	✗	✗	✓	✗
Nahiduzzaman et al. (2025)	✓	✓	✓	✓	✗	✗
Sundararajan et al. (2025)	✓	✓	✓	✗	✗	✗
Rajpoot et al. (2025)	✓	✓	✓	✗	✗	✗
Prakash et al. (2025)	✓	✓	✓	✓	✓	✗
Zheng et al. (2023b)	✓	✓	✗	✗	✗	✗
Zhao et al. (2025)	✓	✓	✓	△	✓	✓
Chel and Poh (2025)	✓	✓	✗	✗	✗	✗
Yang et al. (2024)	✓	✓	✗	✗	✓	✓
Yan et al. (2023)	✓	✓	✗	✗	✗	✗
Xie et al. (2024)	✓	✓	✓	✓	✗	✗

Based on this comparative analysis in Table 7, it can be observed that most of the studies have adopted various *post-hoc* explainability techniques, including Grad-CAM, SHAP, LIME, and attention maps, along with attribution methods to provide explanations regarding the regions of interest. Although there are significant contributions in this area, there are still many research gaps to be filled. For example, there is still limited validation and cross-dataset generalization of brain tumor segmentation and classification solutions. Most of the studies are based on single-dataset scenarios, including BraTS and MRI datasets. Although there are significant attempts to model tumor heterogeneity and complex spatial characteristics, this is still not uniformly integrated into all frameworks. Furthermore, there is limited computational efficiency and real-time feasibility assessment of brain tumor segmentation and classification solutions, considering the increased complexity of deep learning-based architectures, including CNN and Transformer-based frameworks. In addition, only a few studies have incorporated various modalities, including genomic and clinical features, which are essential to understand the biological context of glioma development and progression.

### 4.4 The privacy-preserving nexus: federated and collaborative learning

The overall body of research (Aggarwal et al., 2024; Ren et al., 2025) signals a paradigm shift in decentralized and privacy-preserving machine learning in brain tumor detection research, primarily addressing the conundrum of balancing the requirement for large volumes of data with the stringent legalities around patient confidentiality. By reviewing these research papers collectively, we can identify a shift from conventional centralized architectures to advanced Federated Learning architectures (e.g., FedAvg, FedProx, FedDpu) (McMahan et al., 2017) and Collaborative Convolutional Networks (Raheem et al., 2024; Sivakumar et al., 2025b; Nagoor Kani and Ayothi, 2025; Yahiaoui et al., 2024; Sharma et al., 2025; Sivakumar et al., 2025a), which effectively mitigate the risks associated with sharing raw data. A critical analysis of these research papers reveals that while innovations in machine learning architectures such as Learning Without Forgetting (LWF) (Huang Y. et al., 2024) and integration with Transfer Learning (Albalawi et al., 2024) successfully address the “catastrophic forgetting” and “sufficient data availability” conundrums in multi-center studies, the overall body of research in this domain still grapples with a fundamental trade-off: while the introduction of privacy-preserving noise (e.g., Laplace noise) (Huang L. et al., 2024) can often compromise on the level of granularity required for critical tasks such as MGMT promoter methylation prediction (Atef et al., 2022) or glioma subtyping (Ali et al., 2023; Ren et al., 2025). Moreover, as they are increasingly using Explainable AI (XAI) (Latif et al., 2025) and Hybrid Optimization approaches (Nagoor Kani and Ayothi, 2025; Sivakumar et al., 2025a) for enhanced trust and convergence rate in the results, one of the major limitations of the methodologies discussed in all the articles is the use of fixed datasets like BraTS, indicating perhaps that the current methodologies might not account for the “noise” and high complexity of real-world biological data, where the problem of non-IID is still affecting the results of even the most advanced Federated Learning approaches (Huang Y. et al., 2024; Huang L. et al., 2024; Zahoora et al., 2025; Raheem et al., 2024; Sivakumar et al., 2025a; Ren et al., 2025). A cross-examination analysis of the literature available suggests a very distinct dichotomy between the local optimization measures and the field-level deployment preparation criteria. Instead of merely providing a descriptive evaluation of the methodologies used, this part discusses the trade-off considerations, feature extraction approaches, validation integrity, and clinical constraints for the use of decentralized machine learning in brain tumor detection.

#### 4.4.1 Privacy-preserving taxonomy and topological paradigms

Privacy architecture distribution in current research illustrates the change from separate knowledge transfer mechanisms to optimization loops that involve multiple parties. In line with the classification given in Table 8, decentralization itself determines the trade-off between system vulnerability and complexity.

**Table 8:** Taxonomy of privacy-preserving modalities and architectural bounds across all reviewed studies.

Decentralized paradigm	Architectural and privacy execution	Covered literature reference	Systemic trade-off and critical bottleneck
Sequential/incremental transfer learning	Models are passed sequentially between centers; utilizes frozen layer states or custom Knowledge Distillation Loss (KDL) to guard source domain privacy.	Huang Y. et al., 2024; Aggarwal et al., 2024; Albalawi et al., 2024	Reduces multi-party synchronization overhead but introduces high vulnerability to *Catastrophic Forgetting* under severe domain shifts.
Standard baseline federated learning	Traditional horizontal data partitioning utilizing standard synchronous weight consolidation algorithms (primarily baseline FedAvg).	Lee et al., 2024; Anoosha and Seetharamulu, 2025; Sharma et al., 2024, 2025	Highly susceptible to client gradient divergence and severe utility degradation when applied to non-IID clinical image distributions.
Bias-resilient and regularized FL	Integrates client-filtering frameworks or proximal optimization penalties (FedProx) to bound local weight drift.	Zahoora et al., 2025; Sivakumar et al., 2025a	Minimizes local divergence under non-uniform clustering but significantly increases the local client optimization computational budget.
Advanced dynamic and collaborative frameworks	Utilizes dynamic parameter updating, dual-model streams, comparative learning loops, or intermediate collaborative layers (IPC-CNN).	Huang Y. et al., 2024; Raheem et al., 2025, 2024; Sivakumar et al., 2025b; Pathan et al., 2025	Demonstrates superior resilience to highly skewed distributions; however, it introduces increased algorithmic complexity and high hyperparameter sensitivity.
Specialized downstream analytics pipelines	Integrates privacy-preserving workflows directly with *post-hoc* explainable decision wrappers, federated radiomics, or clinical response metrics.	Ali et al., 2023; Latif et al., 2025; Nagoor Kani and Ayothi, 2025; Atef et al., 2022; Ren et al., 2025	Bridges the gap between raw tensor outputs and clinical interpretability, but remains fundamentally bounded by upstream feature extraction quality.

##### 4.4.1.1 Privacy risks beyond federated data locality

Although Federated Learning avoids direct exchange of raw MRI data, it should not be regarded as a complete guarantee of patient privacy. The model updates exchanged during federated optimization may themselves contain information about local training samples. Gradient leakage or gradient inversion attacks can exploit shared gradients or model updates to reconstruct sensitive characteristics of training images, while model inversion attacks can use model outputs or updates to infer representative features of the underlying data (Zhu et al., 2019). Membership inference attacks pose a different risk by determining whether a particular patient or MRI sample contributed to model training (Shokri et al., 2017; Nasr et al., 2019). These threats are particularly relevant in neuro-oncology because MRI data contain highly sensitive patient-specific anatomical and pathological information. Therefore, privacy protection in federated brain tumor analysis requires safeguards beyond data locality.

Additional privacy-preserving mechanisms can be incorporated to mitigate these risks. Differential Privacy (DP) limits the contribution of individual records by perturbing model updates or gradients with controlled noise, thereby reducing the risk of information disclosure (Abadi et al., 2016; Wei et al., 2020). However, excessive noise may reduce the sensitivity required for detecting subtle tumor characteristics and can introduce a privacy–utility trade-off. Secure Aggregation can further protect client updates by ensuring that the aggregation server receives only an aggregate of participating updates rather than directly inspecting individual client updates (Bonawitz et al., 2017). Consequently, future XAI-FL frameworks for brain tumor MRI analysis should evaluate not only whether raw data remain decentralized, but also the resistance of shared model updates to gradient leakage, model inversion, and membership inference attacks, together with explicit assessment of differential-privacy parameters and secure-aggregation mechanisms.

##### 4.4.1.2 Data heterogeneity and domain shift in federated learning

A major challenge in federated brain tumor MRI analysis is the non-IID nature of institutional data. Differences in scanner manufacturers and field strengths, acquisition protocols, voxel spacing, MRI sequences, patient demographics, disease distributions, and annotation practices can cause substantial variation in the local data distributions across participating institutions. Such heterogeneity can produce client-specific optimization objectives and gradient divergence, causing a globally aggregated model to perform inconsistently across institutions. Domain shift can further arise when a model trained collaboratively across participating centers encounters an unseen institution with different imaging characteristics or patient populations. Therefore, evaluation of federated brain tumor models should extend beyond aggregate global performance and include client-level performance, cross-institutional validation, and explicit assessment of robustness under domain shift. Approaches such as FedProx, adaptive client selection, personalized Federated Learning, and other non-IID-resilient optimization strategies may help reduce the effects of distributional divergence, but their effectiveness should be evaluated under realistic multi-institutional MRI heterogeneity rather than only under artificially homogenized data splits.

#### 4.4.2 Algorithmic backbones and optimization dynamics

Mathematical models are responsible for determining the level of learning capability that can be attained in multi-modal learning. The relationship between feature extraction engines and their respective optimization methods is shown in Table 9. One of the principal distinctions that can be made here is between 3D segmentation models and shallow classification models.

**Table 9:** Feature-extraction backbones, optimization strategies, and interpretability limitations across reviewed federated brain tumor detection studies.

Neural architecture category	Optimization strategy	Covered literature reference	Interpretability/deployment limitation
3D segmentation and modified UNet frameworks	Adam and adaptive weighting schemes	Zahoora et al., 2025; Raheem et al., 2025; Lee et al., 2024; Yahiaoui et al., 2024	High-dimensional 3D feature representations increase computational and communication overhead, while reducing layer-wise interpretability in distributed environments.
Multi-encoder and dense convolutional architectures	SGD- and Adam-based optimization	Raheem et al., 2024	Complex feature fusion pathways may obscure the individual contribution of encoder branches, requiring additional *post-hoc* explainability mechanisms.
Hybrid radiomics and meta-heuristic frameworks	Evolutionary, swarm-based, and hybrid optimization techniques	Ali et al., 2023; Latif et al., 2025; Nagoor Kani and Ayothi, 2025; Atef et al., 2022; Ren et al., 2025	Performance is highly dependent on preprocessing consistency and handcrafted radiomic stability, which may reduce scalability across heterogeneous clinical datasets.

#### 4.4.3 Validation rigor and environmental fidelity

An important problem highlighted through this analysis framework is that of synthetic data segmentation and benchmarking-based validation. This issue can be understood from Table 10, where there is a significant gap between performance results achieved on curated datasets and clinical heterogeneity settings.

**Table 10:** Rigor assessment: clinical validation realism vs. benchmark-driven evaluation strategies.

Validation profile	Data source complexity	Covered literature reference	Critical data integrity gap
Semi-realistic multicenter clinical validation	Heterogeneous datasets collected from multiple clinical institutions with limited protocol harmonization.	Huang Y. et al., 2024; Lee et al., 2024; Ren et al., 2025	Exposes models to scanner variability, inter-observer annotation differences, and heterogeneous acquisition settings that are often absent in curated benchmark datasets.
Cross-silo dataset clustering	Integration of distinct public repositories or institutional cohorts to emulate decentralized silos.	Sharma et al., 2024, 2025; Sivakumar et al., 2025a	Partially captures regional imaging variability; however, standardized preprocessing may reduce the visibility of rare artifacts and edge-case failures.
Artificially partitioned centralized benchmarks	Public repositories (e.g., BraTS) statistically partitioned into simulated federated clients using Dirichlet or distribution-based sampling.	Huang Y. et al., 2024; Zahoora et al., 2025; Aggarwal et al., 2024; Raheem et al., 2025, 2024; Sivakumar et al., 2025b; Albalawi et al., 2024; Anoosha and Seetharamulu, 2025; Ali et al., 2023; Latif et al., 2025; Nagoor Kani and Ayothi, 2025; Atef et al., 2022; Yahiaoui et al., 2024; Pathan et al., 2025	May overestimate generalization performance by insufficiently modeling missing modalities, scanner variability, resolution inconsistencies, and real-world technical noise.

#### 4.4.4 Task-specific boundaries and optimization horizons

Decentralized learning methodologies should eventually be consistent with the demands of clinical precision posed by the applications. The following Table 11 lists some of the key goals in terms of clinical endpoints, corresponding failure points, and methods.

**Table 11:** Clinical objectives, decentralized learning failure points, and methodological optimization strategies.

Clinical objective	Required granularity	Primary limitation	Potential methodological strategy
Brain metastases and intracranial delineation	High voxel-level precision for detecting small and spatially isolated lesions.	Global parameter aggregation may suppress subtle local features, increasing false-negative risk in small lesion regions.	Integrate localized Knowledge Distillation Loss (KDL) and region-aware optimization mechanisms to preserve fine-grained spatial representations.
Whole tumor core segmentation	Accurate delineation of enhancing tumor, edema, and necrotic subregions.	Client drift caused by heterogeneous structural morphology and class imbalance across institutions.	Employ adaptive client selection and dynamically weighted multi-class Dice optimization to stabilize distributed segmentation learning.
Malignant glioma diagnostics and subtyping	Robust extraction of subtle molecular and imaging biomarkers.	Privacy-preserving perturbation may reduce sensitivity to low-contrast imaging and molecular patterns.	Combine regularized federated optimization methods (e.g., FedProx) with stable pretrained feature encoders to preserve discriminative representations.
Explainable AI and advanced clinical prediction	Reliable multimodal feature attribution linked with confidence-aware prediction mechanisms.	*Post-hoc* explainability methods may provide limited fine-grained localization consistency across heterogeneous institutions.	Integrate multimodal attention mechanisms and federated radiomics constraints to improve interpretability robustness across decentralized clinical environments.

As seen from the thorough examination performed within the scope of this section, there is an underlying problem that needs to be addressed—namely, the fact that the necessary structural trade-offs made by any privacy-preserving framework contradict the fine-grained feature resolution needed for safety-critical applications. As demonstrated in Tables 8–11, models which have proven their mathematical privacy guarantees tend to exhibit subpar structural performance. Therefore, future work cannot be limited to assessing homogenized and artificial splits in datasets, but rather should aim at model deployment under realistic non-IID data streams including all missing modalities.

### 4.5 Multimodal and multi-omics frameworks

In brain tumor research, multimodal learning has gained popularity as a result of its capacity for integrating complementary features extracted from different imaging sequences, heterogeneous clinical data, and multi-omics data sources. As opposed to traditional monomodal models, multimodal techniques seek to increase accuracy, improve segmentation quality, enhance interpretability, and boost robustness by fusing distinct feature spaces into one coherent learning framework. Nevertheless, although multimodal methods have achieved impressive results in many recent publications, significant variation exists in aspects related to the fusion mechanism, architectural design, explanation capacity, computational considerations, and experimental validity. This paper therefore provides a critical evaluation of the discussed multimodal and multi-omics research papers by contrasting their methodologies, interpretability techniques, computational challenges, and generalizability problems instead of merely highlighting their results.

#### 4.5.1 Modality fusion paradigms: structural imaging fusion vs. omics-level integration

Another difference between methodologies across the reviewed papers can be found in the way “multi-modal” information is presented. The definition of multimodality is based on multiple MRI sequences, namely, T1, T1ce, T2, and T2-FLAIR in Rawas and Samala (2024), Maqsood et al. (2022), Coupet et al. (2022), and Kesav and Rajini (2024). The approach used to fuse such multimodal data is predominantly focused on images and utilizes CNNs to fuse spatial and structural information from these sequences. One of the more technically advanced works that can be found in the literature review is Coupet et al. (2022). This paper provides focused research into the effects of different MRI sequence-network pairs. The results have shown that the choice of a particular combination determines the performance level significantly and that T2-FLAIR proved to be the best modality used individually. Moreover, excluding certain pairs of MRI sequences demonstrated that their fusion adds complementary information. On the other hand, Zhang et al. (2025) transcend the realm of structural imaging by introducing the use of MOFNet for multi-omics classification of cancer subtypes. Unlike spatial fusion approaches which capitalize on the notion of anatomical continuity, MOFNet uses genomics, transcriptomics, and epigenomics data via Similarity Graph Pooling (SGO) and decision-level fusion using the View Correlation Discovery Network (VCDN). Unfortunately, despite the novel design of the proposed architecture, Zhang et al.'s (2025) study is only confined to three TCGA datasets without showing how robust the model would be among other clinical samples or other omics modalities. As such, although the model introduces an interesting expansion of multimodal fusion, its practicality remains unproven.

#### 4.5.2 Architectural sophistication vs. computational efficiency

The studies reviewed here show an evident compromise made between the architectural sophistication of the models and the ability to computationally optimize them. Coupet et al. (2022) use several complex pretrained models, specifically, VGG19, InceptionV3, ResNet50, DenseNet169, EfficientNet-B6, and UNET-3D. The described architectures are powerful from the viewpoint of representation learning; however, their optimization is rather problematic. Particularly, the authors note problems with the performance of UNET-3D due to convergence issues and local minima. Additionally, they report significant overfitting without using dropout regularization, where some models demonstrate close to perfect training accuracy but fail at validation. It adds validity to the results presented in the paper since the authors do not ignore limitations associated with the complexity of deep neural networks for medical data. Kesav and Rajini (2024) emphasize simplicity of computation via their Reduced Complexity Spatial Fusion CNN (RCSF-CNN). Unlike the end-to-end approach for learning features, their architecture incorporates Discrete Orthogonal Stockwell Transform (DOST), in addition to hand-engineered features like entropy, variance, and contrast, in classification. Likewise, Maqsood et al. (2022) have used a light-weight variant of the MobileNetV2 network with multiclass SVM classifiers and feature selection using entropy. Despite the very high accuracy levels of these simpler frameworks being well above 98%, it would be prudent to treat these assertions with some caution. With respect to Kesav and Rajini (2024), the design of the experiment relies on a very limited set of data that significantly raises the chances of overfitting and skewed accuracy levels. Thus, such a high level of accuracy achieved by the authors might not represent generalization but rather a case of memorizing the limited dataset used. In addition, even as complexity simplification reduces neural net size, it increases computation during data preprocessing and feature engineering.

In order to further reinforce the comparative synthesis of multimodal and hybrid neuro-oncological AI models, Table 12 provides a comparative evaluation of the discussed architectures in relation to their architectural approach, validation method, incorporation of explainability, translational challenges, and readiness for clinical deployment.

**Table 12:** Cross-study comparative synthesis of multimodal and hybrid neuro-oncology AI frameworks.

Study/framework	Data modality	Architectural strategy	Validation setting	Explainability integration	Observed technical limitation	Critical translational insight
ChatGPT-CNN multimodal brain tumor analysis (Rawas and Samala, 2024)	Multi-modal MRI (BraTS)	CNN + conversational explainability interface	Single benchmark dataset evaluation	ChatGPT-assisted interpretation interface	Limited external validation and restricted clinician usability evaluation	Interpretability enhancement remained largely interface-driven rather than biologically validated, limiting real-world clinical trust despite strong segmentation performance
MOFNet: multi-omics fusion framework (Zhang et al., 2025)	Multi-omics + genomic cancer datasets	GCN-based supervised multi-omics fusion with VCDN integration	Limited TCGA cancer-type evaluation	Graph-based feature interpretation	Scalability concerns under large label spaces and limited biological validation	Although multimodal omics fusion improved subtype classification, translational reliability remained constrained by weak biomarker-level validation and limited dataset diversity
MobileNetV2 + DNN + M-SVM hybrid brain tumor classification framework (Maqsood et al., 2022)	Multi-modal MRI (BraTS + Figshare)	Transfer learning + hybrid CNN-SVM classification	Dual benchmark dataset validation	Feature-selection-driven interpretability	2D-only implementation and computationally expensive feature selection	Hybrid architectures improved classification separability and multiclass prediction accuracy, but scalability toward volumetric MRI workflows remained limited
Multi-sequence glioma deep learning framework (Coupet et al., 2022)	T1, T1ce, T2, and FLAIR MRI sequences	Transfer learning using ResNet50, DenseNet, InceptionV3, EfficientNet, and UNET-3D	Internal validation with multi-sequence comparison	Layer-wise relevance propagation and deep feature visualization	Overfitting under limited data, modality sensitivity, and local minima instability in 3D models	The study demonstrated that multimodal MRI fusion substantially improves contextual tumor characterization; however, performance stability remained highly dependent on MRI sequence selection and data availability
Cross-study multimodal trend	MRI + radiomics + omics integration	Hybrid multimodal fusion and transfer learning paradigms	Predominantly retrospective benchmark datasets	Predominantly *post-hoc* explainability frameworks	Weak external validation and high computational dependency	Most multimodal neuro-oncology frameworks improved predictive accuracy and feature richness, yet remained constrained by poor clinical-scale validation, computational complexity, and limited biological interpretability
Cross-study generalization gap	Heterogeneous MRI and omics distributions	Complex hybrid and multi-branch architectures	Limited multi-center prospective evaluation	Visualization-based explainability dominance	Sensitivity to dataset heterogeneity and domain shift	Despite promising multiclass classification performance, current multimodal frameworks remain insufficiently robust for routine clinical deployment under heterogeneous imaging environments

#### 4.5.3 Explainability and clinical interpretability challenges

sExplainability remains one of the most important unresolved issues across the reviewed studies. Kesav and Rajini (2024) apply the Class Activation Mapping (CAM) technique for pointing out relevant tumor locations, and Coupet et al. (2022) rely on three techniques including LRP, Deep Taylor decomposition, and Guided Backpropagation for visualizing the feature relevance. In this regard, both methods increase explainability because they show the relevance of predictions to particular image areas. Nevertheless, due to their mathematical complexity, visualization techniques need further explanation, and it is not easy to provide a clinical understanding of the generated explanations through the heatmaps. To solve this problem, Rawas and Samala (2024) implement a novel approach for linking the CNN model and ChatGPT to create a conversational explanation tool based on human-language input and output. Although this method increases usability and also interactive experience, the study fails to prove causality between the chatbot responses and the operations of the neural network. This introduces an important clinical concern. Large language models can generate fluent but potentially inaccurate explanations, creating a risk of overreliance by clinicians. Therefore, although the integration of generative AI is innovative, the interpretability benefits remain preliminary and require stronger validation before clinical deployment.

#### 4.5.4 Data scarcity, validation limitations, and generalizability

One of the consistent drawbacks found in almost all reviewed papers is the lack of validation scale and poor proof of clinical applicability. This issue is addressed by Kesav and Rajini (2024). Despite the fact that the model is described as having a 98.99% accuracy and a specificity of 99.76%, it was tested on just 32 patients and evaluated through just five images for assessing features. A study of this small size cannot provide sufficient statistical evidence to claim robustness or applicability. Coupet et al. (2022) clearly state the problem of overfitting with regard to limited training data in medicine. The authors prove the fact that deep learning approaches may fail under such conditions without proper regularizing procedures. Their work indicate more methodologically reliable than papers which provide extremely accurate results without addressing validation concerns. In similar manner, the study of Rawas and Samala (2024) validates their framework using the BraTS dataset alone, whereas that of Zhang et al. (2025) is based solely on the use of TCGA samples. Despite being considered well-established benchmark datasets, both BraTS and TCGA datasets are heavily curated and thus cannot possibly capture the complexity of the real-life scenario encountered at hospitals. Moreover, one of the critical limitations found in Coupet et al. (2022) involves converting MRI data from 16-bit voxel representation to 8-bit PNG images to make them compatible with pretrained 2D CNN architectures. Although the performance losses associated with such an approach appear minor, this method still causes information loss in the input MRI data. Table 13 provides a brief but critical comparison of the studied multimodal and multi-omics analyses according to the methodologies utilized, interpretability methods applied, achieved performances, and main generalizability issues. In contrast to the summary of findings reported by various researchers, Table 13 demonstrates common problems associated with data dependence, computationally intensive algorithms, explainability, and clinical robustness.

**Table 13:** Critical analytical comparison of multimodal and multi-omics brain tumor studies.

Paper	Problem domain	Framework	Data/modality	XAI method	Performance	Critical limitations
Rawas and Samala (2024)	Brain tumor segmentation and clinical communication	Transfer learning CNNs (VGG, ResNet, DenseNet) integrated with chatbot support	BraTS multi-modal MRI	ChatGPT-assisted explanations	DSC = 0.89, F1 = 0.90	Single-cohort evaluation and limited validation of chatbot-based explanations.
Zhang et al. (2025)	Cancer subtype classification using multi-omics	MOFNet with GCN-based SGO and VCDN fusion	TCGA multi-omics datasets	Graph pooling interpretability	Accuracy up to 89.36%	Validated only on limited TCGA cohorts with insufficient biological verification.
Maqsood et al. (2022)	Brain tumor detection and classification	17-layer DNN + MobileNetV2 + LSTM + M-SVM	BraTS 2018 and Figshare MRI	No explicit XAI	Accuracy up to 98.92%	Restricted to 2D slice processing and computationally intensive feature selection.
Coupet et al. (2022)	Glioma classification	CNN ensemble with UNET-3D	Multi-sequence MRI	LRP, Deep Taylor, Guided Backpropagation	Accuracy up to 0.8638	Overfitting risk under limited data and reduced precision from 8-bit image conversion.
Kesav and Rajini (2024)	Computationally efficient tumor classification	RCSF-CNN with DOST-based feature extraction	Brain tumor imaging data	CAM visualization	Accuracy = 98.99%	Very small dataset limits statistical reliability and generalizability.

The ratings in Table 13 were derived by applying the operational criteria in Table 14 to the evidence synthesized across the reviewed studies within each methodological category. These category-level ratings represent the predominant evidence pattern and do not imply that every study within a category received the same rating. In conclusion, while the reviewed literature suggests significant advancement in multimodal learning and fusion methods, the majority of papers are still largely characterized by a lack of external validation, dataset reliance, and limited testing on more clinically realistic samples. Therefore, much of the impressive performance metrics observed in the literature can only be viewed as experimental results.

**Table 14:** Operational criteria used for qualitative ratings in comparative tables.

Dimension	✓strong/full	△Partial/limited	×Weak/absent
XAI support	Explicit XAI or intrinsically interpretable mechanism with a clearly described explanation process	Basic or *post-hoc* explanation method is present, but explanation depth or validation is limited	No XAI mechanism or interpretability evidence is reported
Validation rigor	Independent test set and/or external validation, preferably with cross-validation or multi-center evaluation	Internal train/test split or cross-validation without external or independent clinical validation	Single-split evaluation, insufficient validation description, or no meaningful validation evidence
Data generalization	Evaluation across independent datasets, institutions, acquisition settings, or explicit domain-shift/generalization testing	Multiple datasets or robustness analysis is reported, but external or cross-institutional validation is limited	Evaluation is restricted to a single curated dataset with no meaningful generalization analysis
Computational Burden	Computational requirements are explicitly evaluated and reported as manageable, or the architecture is demonstrably lightweight/efficient	Moderate architectural or computational complexity is evident, but deployment cost is not fully characterized	High computational, memory, communication, or architectural complexity is evident without adequate efficiency or deployment analysis
Clinical readiness	Evidence of external or clinical validation together with clinically relevant evaluation, calibration, uncertainty, or workflow assessment	Promising experimental performance with some clinically relevant evaluation, but without sufficient external or prospective validation	Only benchmark performance is reported, with no meaningful clinical validation or workflow/deployment evidence
Clinical interpretability	Explanations are quantitatively or clinically validated and linked to anatomically or biologically meaningful features	Visual or *post-hoc* explanations are provided but have limited clinical or biological validation	No meaningful interpretability mechanism or validation is reported
Multimodal integration	Multiple MRI sequences and/or complementary clinical, radiomic, genomic, or pathological modalities are explicitly integrated	Limited multimodal information or partial fusion is used	Single modality or no meaningful multimodal integration

## 5 Discussion

### 5.1 Critical synthesis of trustworthiness, generalizability, and clinical translation challenges

One of the key insights derived from the above analysis is that AI research in brain tumor detection is moving beyond simple accuracy-driven learning models to more trustworthy intelligent systems for clinical use. Although previous research efforts focused mostly on achieving high accuracy results by using neural networks for segmentation purposes and optimizing Dice-score values, today's research tends to pay more attention to explainability, data privacy protection, multimodal reasoning capabilities, and distributed learning strategies. As depicted in our framework of the systemic architecture transition (Figure 9), it appears that improvements in architectural design have been outpacing those in other areas significantly.

**Figure 9:**
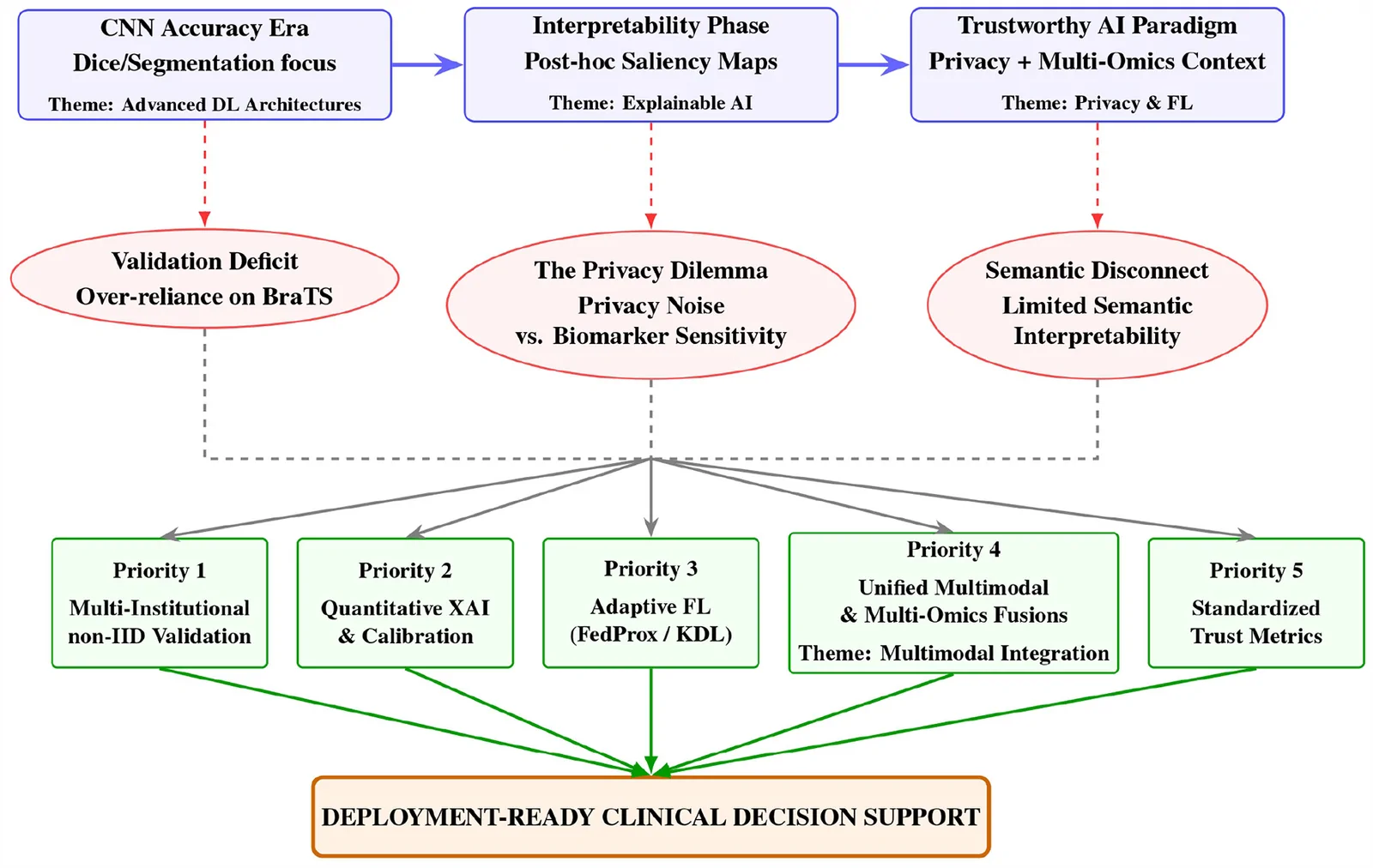
Conceptual evolution of brain tumor MRI analysis from accuracy-driven CNN systems toward trustworthy AI frameworks, highlighting major deployment bottlenecks and strategic research priorities required for trustworthy AI-based brain tumor detection and clinical decision support.

Among the clear trends that have emerged from reviewing the literature in this area is the reliance on *post-hoc* interpretability frameworks such as Grad-CAM, SHAP, LIME, LayerCAM, Guided Backpropagation, and attention visualization. These techniques have been incorporated extensively into CNNs, Transformers, and multimodal architectures to provide visual indications of regions or features associated with model predictions. However, the review indicates that the availability of an XAI visualization does not necessarily establish the reliability or clinical validity of the explanation. Quantitative evaluation of explanation quality was uncommon compared with the extensive reporting of predictive performance. In many studies, XAI was presented primarily through qualitative heatmaps, saliency maps, attention maps, or feature-attribution visualizations, without standardized quantitative assessment of explanation reliability or agreement with clinically meaningful regions. Domain-expert validation was also limited, with explicit radiologist-based assessment of explanations identified in only a small number of studies. Thus, XAI visualization was distinguished from validated interpretability, with quantitative explanation assessment and expert evaluation considered stronger forms of evidence for establishing clinically meaningful explanations. Moreover, another issue identified through the literature review is the growing gap between architectural sophistication and biological plausibility. Architectures based on Transformer models and hybrid architectures have demonstrated improved capacity to represent contextual information in volumetric MRI analysis; however, increasing architectural complexity can make it more difficult to establish the biological relevance of the resulting feature-attribution patterns. While several studies based on vision Transformers and hybrid CNN-Transformer models have demonstrated strong performance in segmentation and classification tasks, their patch-token attention mechanisms did not always provide clear clinical semantic relevance.

Clinical validation and external generalization were also examined across the reviewed studies using the study-level evidence matrix. Of the 146 included studies, 33 were explicitly identified as reporting external validation or independent external testing; however, these evaluations were heterogeneous and included both independent clinical cohorts and cross-dataset benchmark testing. Multi-institutional and cross-institutional evaluation was reported in a smaller subset of studies, including studies using data from independent institutions or multi-center cohorts. Direct evaluation of model explanations against radiologist assessment was uncommon, with explicit radiologist-based assessment identified in only a small number of studies. Prospective clinical evaluation was also rare; one study explicitly reported prospective evaluation of 50 consecutive cases, whereas several other studies identified prospective validation as a future requirement rather than as a completed evaluation. Therefore, external testing should be distinguished from prospective clinical validation, and multi-institutional or cross-dataset evaluation alone does not establish clinical readiness. Overall, the evidence indicates that most reported performance remains based on retrospective cohorts, public benchmarks, or internal validation, with limited evidence from independent clinical populations and prospective clinical deployment.

Moreover, the review emphasizes the methodological gap between the centralization of explainability studies and the development of privacy-preserving approaches to collaborative learning. While most of the relevant research is dedicated to the topic of explainability, less attention is paid to the topics of Federated Learning (FL), differential privacy, or decentralization of the optimization process. The problem under consideration highlights one of the critical deployment issues faced by clinical brain tumor detection. Since the disease under study implies multi-institutional learning because of the rarity and diversity of patients' cases, centralized training is limited by GDPR, HIPAA, and the principle of institutional data sovereignty. One important trade-off that emerges frequently in the FL body of work is the one between protecting privacy and ensuring feature sensitivity. Methods like differential privacy and adding noise improve privacy protection but can potentially hinder the identification of fine imaging markers required to classify lesions with poor contrast, predict MGMT methylation, and subtype gliomas. This trade-off exacerbates when considering a Federated Learning approach on non-IID institutionally distributed data due to variations in scanner configurations, voxel spacing, missing modality information, and variable annotations. While methods like FedProx, dynamic collaboration schemes, adaptive client selection strategies, and knowledge distillation loss (KDL) can help mitigate weight drifts and forgetting problems, they complicate the optimization process and increase communication costs. The final major issue revolves around the overly optimistic use of benchmarks like BraTS and TCGA. While these benchmarks are crucial in any study due to the need to compare methodologies, they do not capture the reality seen in clinics due to their curate nature and inability to capture the noisiness and inconsistency of actual medical imaging as well as demographic variability. The studies under review claim extraordinarily high metrics such as accuracies of over 98% or dice scores nearing perfection while using very few patients, Federated Learning partitions, or simply one dataset at best. Moreover, the findings suggest that there is a growing need to learn in multimodal and multi-omics settings for representing the biological variability of brain tumors. Indeed, image sequence fusion of T1, T1ce, T2, and FLAIR images has been widely used in the segmentation and classification of brain tumors since different images reveal different features. Furthermore, novel paradigms based on genomics, transcriptomics, and radiomics try to go beyond anatomy and develop biologically-driven predictive models. Yet, despite these advancements, the current multimodal models are computationally costly and rely significantly on properly curated pre-processing pipelines. Additionally, very few multimodal studies have been externally validated. The review also highlights a basic conflict between the computational efficiency and clinical reliability. Efficient architectural designs, together with hand-crafted feature engineering strategies, tend to produce highly accurate results with minimal computation effort; nevertheless, these approaches mostly utilize small datasets and simple evaluation setups. By contrast, huge Transformer-based models, along with large-scale 3D multimodal frameworks, offer superior contextual learning capabilities but are characterized by heavy computational overhead, non-interpretable design, excessive memory usage, and non-real-time deployment capability. Consequently, balancing all four factors simultaneously may remain challenging for future brain tumor detection applications. One more important finding concerns the non-existence of established evaluation frameworks for trustworthy AI in the domain of brain tumor detection. The reviewed literature reveals significant differences among validation strategies, explainability measures, data heterogeneity, uncertainty quantification, and privacy benchmarks. Although performance metrics like the Dice score, Hausdorff distance, sensitivity, and specificity are usually evaluated, very few papers measure explanation accuracy, calibration, domain shift robustness, or robustness at deployment time. Likewise, many Federated Learning approaches evaluate their convergence on synthetic data client splits, rather than hospital data splits that would be heterogeneous in reality. It is essential to note that the reviewed literature reveals a gradual trend from “high-performance AI” toward trustworthy AI for clinical deployment. While early models based mainly on CNNs focused on high segmentation accuracy, current literature is moving toward approaches involving interpretability layers, multimodal reasoning, uncertainty considerations, and privacy-aware cooperation. Recent advancements including the introduction of Vision Transformers, State Space Models (Mamba), and multimodal attention-based reasoning imply that next generation brain tumor detection using AI would be context-aware and biologically-integrated models as opposed to purely spatial feature extractors.

The comparative analysis of the different categories presented in Table 15, emphasizes a crucial difference in the research landscape. Although Multimodal and XAI-driven models have reached high levels of interpretability, they are often limited by considerable computational costs and a lack of data set diversity. On the other hand, FL embodies the sole data diversity research category, although often at the expense of performance and depth. However, it is worth mentioning that the absence of standardized clinical validation in all categories is the major limiting factor for practical application. However, although the literature seems promising, it reveals that this area is currently in transition but not yet clinically matured. The fact that most articles use retrospective datasets, curated public benchmarks, and internally validated models implies that prospective validation, clinical integration, uncertainty-aware diagnoses, and robustness across different institutions are underexplored research avenues.

**Table 15:** Comparison of methodological categories in brain tumor analysis.

Category	Interpretability	Dataset diversity	Computational cost	Clinical validation
CNN models	×	×	✓	×
Transformer models	△	×	×	×
XAI models	✓	×	△	×
Federated Learning (FL)	△	✓	△	×
Multimodal models	✓	△	×	×

The comparative analysis of the reviewed studies reveals that none of the considered research paradigms allows meeting all criteria of transparency, privacy protection, robustness, computational efficiency, and clinical applicability (Table 16). Classical deep learning models ensure high prediction quality but are poorly interpretable. Explainable AI models increase the level of interpretability but are rarely subjected to strict biological validation. Federated Learning reduces direct exposure of raw patient data but does not inherently eliminate privacy risks, as shared model updates may remain vulnerable to gradient leakage, model inversion, and membership inference attacks. Transformer and Mamba models enable good representation learning but are computationally expensive and require validation on large samples. Multi-modal frameworks allow complete tumor characterization due to integration of diverse data sources; however, they create significant problems related to data standardization and workflow organization. Overall, this review indicates that future applications of AI in neuro-oncology would result from the combination of explainability, privacy protection, improved representation learning, and multi-modality rather than from one specific paradigm.

**Table 16:** Comparative analysis of different paradigms in neuro-oncology AI.

Paradigm	Primary objective	Major strength	Key limitation	Clinical carrier
Deep learning frameworks	Classification and segmentation	High predictive performance	Black-box decision making	Limited clinician trust
Explainable AI	Model transparency	Improved interpretability	Limited biological validation	Clinical acceptance
Federated Learning	Privacy preservation	Multi-institutional collaboration	non-IID data challenges	Deployment scalability
Advanced architectures	Long-range representation learning	Superior contextual understanding	High computational complexity	Resource requirements
Multi-modal learning	Focused tumor characterization	Rich biological representation	Data fusion complexity	Workflow integration

Table 17 presents a global synthesis of the major research paradigms in trustworthy AI-based brain tumor detection. For each paradigm, the table summarizes its primary strengths, key limitations, emerging research directions, and current level of clinical readiness. This synthesis provides a focused overview of the evolution of the field while highlighting the remaining challenges and future opportunities for clinically deployable trustworthy AI systems.

**Table 17:** Global cross-domain synthesis of trustworthy brain tumor detection using AI frameworks.

Research paradigm	Primary strength	Major limitation	Emerging research direction	Clinical readiness
CNN and transfer learning frameworks	High classification accuracy and efficient feature extraction using pretrained architectures	Dataset dependency, weak external generalization, and *post-hoc* explainability limitations	Lightweight domain-adaptive architectures with biologically grounded explainability	Moderate
Explainable and interpretable AI models	Improved transparency through Grad-CAM, SHAP, LIME, and intrinsic explanation frameworks	Lack of quantitative clinician validation and inconsistent explanation reliability	Clinically validated causal explainability and uncertainty-aware XAI systems	Moderate
Hybrid, radiomics, and multimodal systems	Enhanced prognostic prediction through integration of imaging, genomics, and clinical metadata	High feature redundancy, segmentation sensitivity, and computational complexity	Radiogenomic foundation models with standardized multimodal fusion pipelines	Moderate–High
Advanced robust learning paradigms	Improved adaptability under limited labels, missing modalities, and domain shifts	High inference cost, GAN artifact risks, and persistent benchmark dependency	Foundation-model pretraining and domain-generalizable uncertainty-calibrated AI	Emerging
Clinical workflow and prognostic systems	Integration of AI into prognosis prediction, surgical planning, and workflow automation	Retrospective cohort dependency, scalability limitations, and regulatory constraints	Prospective clinician-in-the-loop validation and scalable deployment frameworks	High Potential
Privacy-preserving and federated learning	Secure decentralized collaboration without direct patient data sharing	Performance degradation under non-IID distributions and strong privacy constraints	Trustworthy federated multimodal learning with adaptive privacy-preserving optimization	Emerging
Hybrid optimization-driven brain tumor detection	Improved feature separability and survival prediction through optimization-driven learning	Increased architectural complexity and limited reproducibility across institutions	Computationally efficient optimization-aware trustworthy AI systems	Moderate

### 5.2 Dataset landscape in neuro-oncology AI

The reviewed literature shows substantial reliance on publicly available benchmark datasets, particularly the Brain Tumor Segmentation (BraTS) challenge datasets. Among the 146 included studies, BraTS was reported in 63 studies (43.2%), making it the most frequently reported dataset, followed by clinical cohorts in 20 studies (13.7%), TCGA in 13 studies (8.9%), and Figshare in 11 studies (7.5%). For the dataset-frequency analysis, studies were coded according to the principal recurring dataset categories identified in the study-level evidence matrix. Because individual studies could report multiple datasets, dataset frequencies were not mutually exclusive and were therefore not expected to sum to 146, as shown in Figure 10.

**Figure 10:**
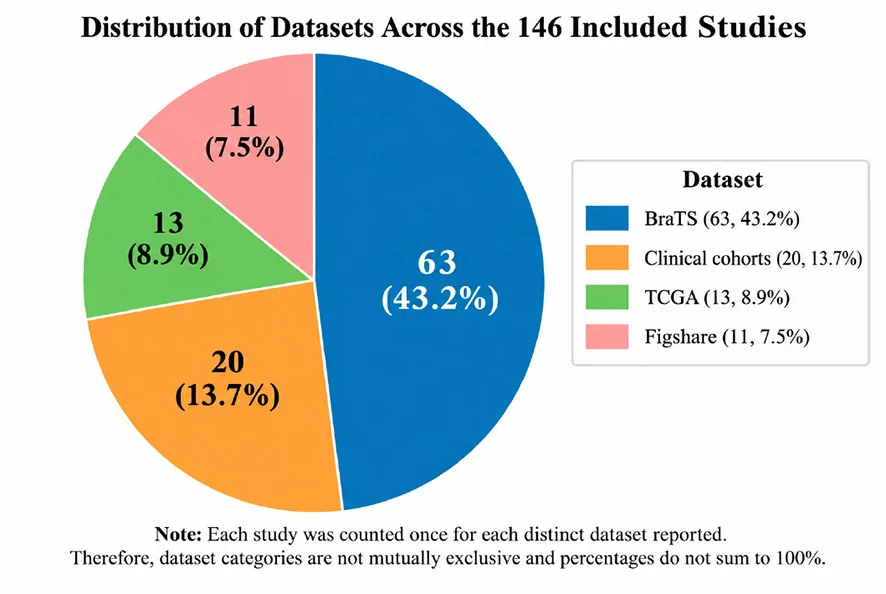
Distribution of datasets reported across the 146 included studies. BraTS was the most frequently reported dataset, occurring in 63 studies (43.2%). Because individual studies could use multiple datasets, dataset frequencies are not mutually exclusive and may exceed the number of included studies when summed across categories.

On the other hand, The Cancer Genome Atlas (TCGA) datasets are mainly used in research related to molecular characterization, survival prediction, and multi-omics studies. Unlike BraTS, which concentrates on the imaging-based tumor delineation, TCGA provides complementary genomic, transcriptomic, and clinical data, allowing the design of predictive models. Less number of research studies use Figshare datasets, Institutional cohorts, and multi-center clinical datasets. These datasets are primarily used for tumor classification and diagnostic decision support. While these datasets do add significant diversity to imaging features and patients, their use is considerably lower compared to BraTS. This difference emphasizes the reliance of the scientific community on only a few benchmarks. Distribution of the observed dataset also highlights an important issue with regard to the neuro-oncology AI literature. Although several papers have documented strong performance on benchmark datasets, the study-level evidence matrix indicates that external testing remains heterogeneous, with some studies using independent datasets or clinical cohorts while others relying primarily on cross-dataset benchmark evaluation. Consequently, external testing should be distinguished from independent clinical validation, and evidence for generalization across institutions and patient populations remains limited. While the dataset distribution highlights the resources most frequently adopted by the neuro-oncology AI community, dataset popularity alone does not reveal how these resources contribute to different research objectives. In practice, individual datasets exhibit strong specialization toward particular tasks, ranging from tumor segmentation and classification to molecular characterization and prognostic modeling. Therefore, a dataset–task relationship analysis was conducted to identify the functional roles of major datasets across the neuro-oncology AI pipeline.

### 5.3 Dataset–task relationship in neuro-oncology AI

Connection between datasets and downstream clinical goals gives us an understanding of how AI research in neuro-oncology is developing now. Datasets are not universal resources; most of them show very specific usage. It can be seen from Figure 11, that some of datasets play the role of benchmark for imaging problems, while others are mainly used for molecular profiling and survival predictions.

**Figure 11:**
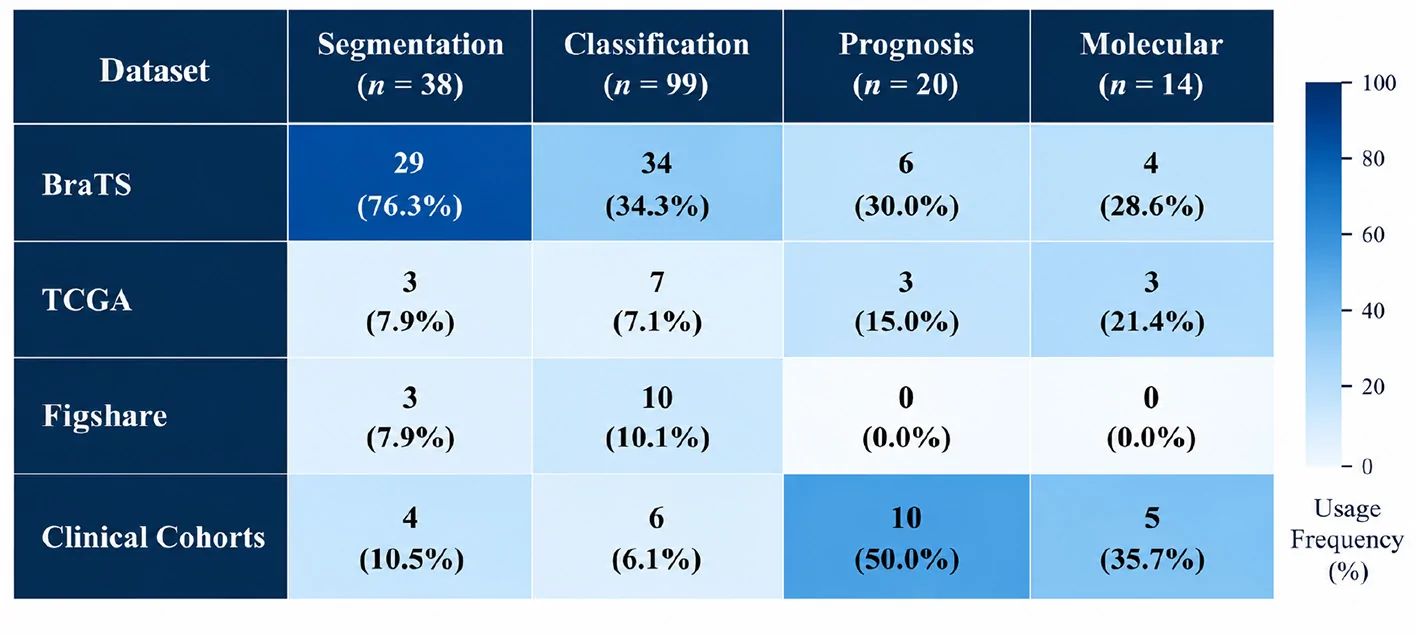
Heatmap of dataset utilization across major neuro-oncology AI tasks.

The specialization of datasets in relation to neuro-oncology AI tasks is evident from Figure 11. Using the final set of 146 included studies, BraTS was reported in 29 of 38 segmentation studies (76.3%) and 34 of 99 classification studies (34.3%). BraTS was also reported in 6 of 20 prognosis studies (30.0%) and 4 of 14 molecular studies (28.6%). TCGA was reported in 3 segmentation studies (7.9%), 7 classification studies (7.1%), 3 prognosis studies (15.0%), and 3 molecular studies (21.4%). Figshare was reported in 3 segmentation studies (7.9%) and 10 classification studies (10.1%), while clinical cohorts were reported in 4 segmentation studies (10.5%), 6 classification studies (6.1%), 10 prognosis studies (50.0%), and 5 molecular studies (35.7%).

For each task category, percentages are calculated using the total number of studies assigned to that task as the denominator. Within each task category, a study was counted once for each distinct dataset it reported, regardless of repeated use of that dataset across experiments. Because studies could address multiple tasks, studies were counted in each applicable category; therefore, task-specific denominators may differ and are not mutually exclusive. The study-level assignments underlying these dataset–task counts are provided in Supplementary File 2 (Dataset–Task Contribution Audit).

The specialization in data sets and application areas seen directly affects the choice of the performance criteria to be used. Neuro-oncology applications have widely varying performance criteria ranging from overlaps in segmentation tasks to discrimination, calibration, and survival analysis in prediction applications. This means that understanding the performance criteria landscape is important in order to understand what results mean.

### 5.4 Evaluation metrics landscape

For the evaluation-metric analysis, a representative subset of 61 studies was used because these studies provided sufficiently explicit and comparable information on the evaluation metrics reported in the full-text analysis. The denominator for the percentages in Table 18 is therefore 61 rather than the total 146 included studies. The subset was selected from the included studies for which metric information could be reliably extracted and categorized into the metric families shown in the table. Each metric family was counted independently at the study level; therefore, a single study could contribute to multiple metric categories when it reported multiple evaluation metrics. For clinical utility, a study was coded when Net Benefit or Decision Curve Analysis was explicitly reported in the study-level evidence matrix, either under reported metrics or validation strategy. Consequently, the percentages in Table 18 are not expected to sum to 100%.

**Table 18:** Distribution of evaluation metrics reported in the 61-study evaluation subset.

Metric family	Metrics included	Number of studies*	Percentage (%)
Accuracy-based	ACC, classification accuracy, Pixel accuracy	45	73.8
Sensitivity/recall	SEN, recall, TPR	18	29.5
Specificity	SPE	15	24.6
Precision	Precision, PPV	14	23.0
F1-score	F1, BF1	17	27.9
AUC-based	AUC, ROC-AUC, IAUC, PR-AUC	15	24.6
Dice-based	DSC, Dice	17	27.9
Boundary metrics	HD, HD95	4	6.6
IoU-based	IoU	1	1.6
Calibration metrics	Brier score, ECE, MCE, NLL	4	6.6
Survival metrics	C-index, Spearman Correlation	3	4.9
Clinical utility metrics	Net benefit, decision curve Analysis	2	3.3
Uncertainty metrics	Uncertainty correlation	1	1.6

Percentages are calculated using 61 studies as the denominator. Metric categories were counted independently at the study level; therefore, studies reporting multiple metrics may contribute to multiple categories and the percentages do not sum to 100%.

The 61-study subset was selected because these studies provided sufficiently explicit and comparable information on the evaluation metrics used. For the remaining included studies, metric information was either not reported in sufficient detail or could not be reliably categorized into the predefined metric families. Therefore, only studies with reliably extractable and comparable metric information were included in the evaluation-metric analysis. The 61-study denominator was applied consistently to all metric frequencies and percentages in Table 18 and Figure 12.

**Figure 12:**
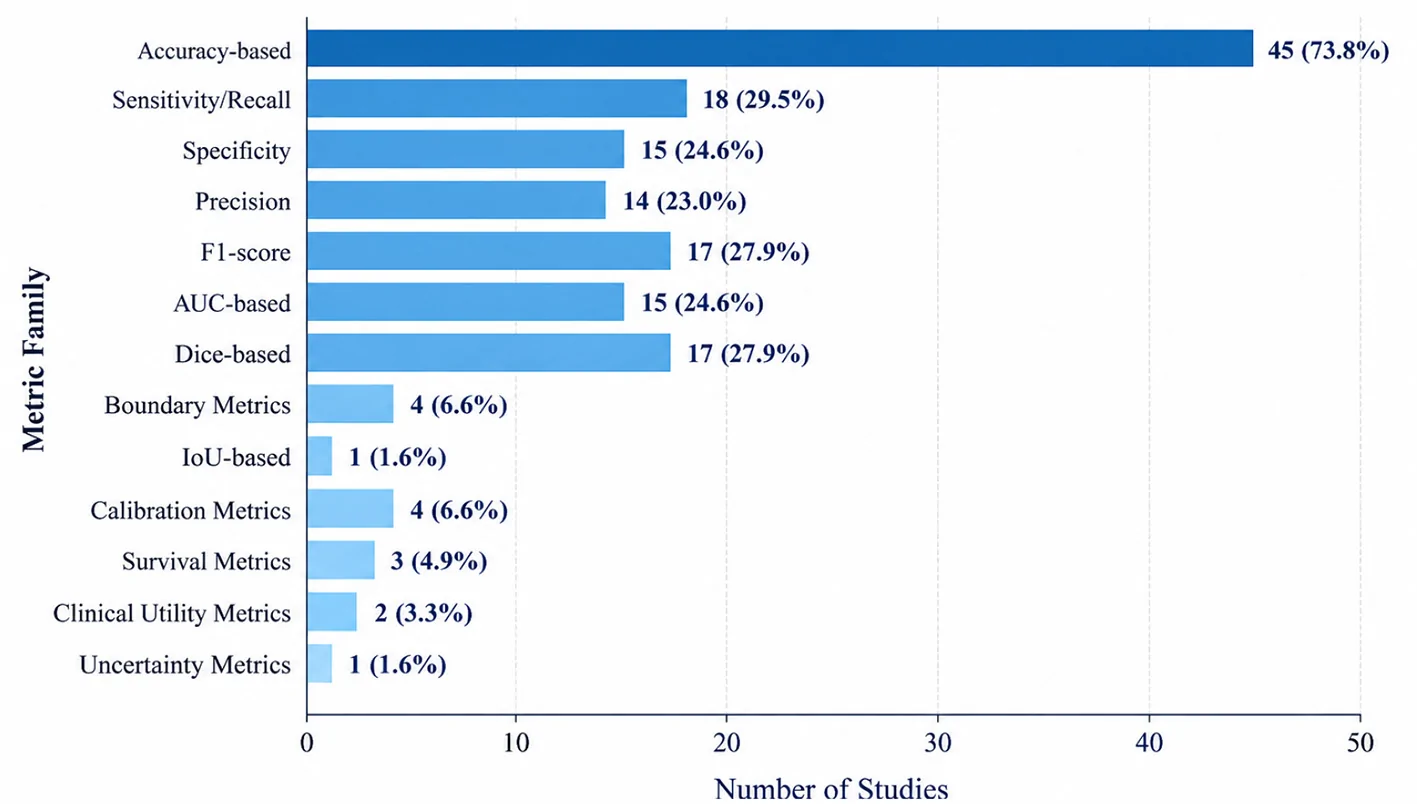
Distribution of evaluation metrics reported across the included brain tumor detection studies. Bars indicate the number of studies reporting each metric family.

Figure 12 and Table 18 together show an evident tendency to use only a few performance-based metrics when conducting evaluation studies. The accuracy metric is used in 45 papers, which is much more than any other evaluation criterion. Moreover, based on the cumulative distribution, one can conclude that only a small number of metrics, such as accuracy, sensitivity, Dice score, F1-score, specificity, AUC, and precision, make up the largest share of all evaluations. Calibration, uncertainty measurement, survival-based metrics, and clinical usefulness evaluation account for only a small part of the reviewed literature. When it comes to classification, accuracy continues to be the most commonly reported metric despite all the drawbacks inherent in such an approach. Multiple studies report classification accuracies higher than 95%, and some approaches reach or exceed the 99% level. Although it is quite clear that there has been considerable methodological progress in the field, this tendency of getting close to ceiling levels of accuracy suggests that the benchmark evaluation has reached its saturation point. Most segmentation algorithms have been evaluated using Dice Similarity Coefficient (DSC), followed by the relatively rare use of Hausdorff Distance (HD95). While DSC serves as a good metric for volumetric overlap between ground-truth and predictions, it lacks the ability to measure border matching or relevant contour deviations. Importantly, very few studies used border-aware metrics such as HD95, which emphasizes the predominance of volumetric overlap measures over anatomically accurate ones. AUC-based metrics were reported in about one-fourth of the studied papers, mainly in diagnosis and prognosis tasks. In comparison with accuracy, AUC allows a more consistent evaluation of the discriminatory power irrespective of different decision thresholds, and thus, is a better fit for imbalanced clinical data. However, its usage is significantly less prevalent compared to classification metrics. The least adopted category among all the metrics mentioned can probably be considered the reliability-based metrics, namely calibration measures (such as Brier Score, ECE, MCE, NLL) which were found in less than 7% of the papers reviewed. Similarly, uncertainty quantification metrics were very rarely used despite being crucial for risk-sensitive clinical decisions. These results imply that the predominant part of the modern neuro-oncology AI frameworks is currently optimized not in terms of confidence reliability but predictive accuracy.

There was an even bigger discrepancy in case of clinical utility assessment. Only few studies considered using Decision Curve Analysis (DCA) or Net Benefit calculations, or similar metrics of the practical clinical value. As expected, survival-oriented metrics such as the concordance index (C-index) and integrated AUC (IAUC) were mostly used in case of prognostic researches and underrepresented in comparison with classification/segmentation metrics. In summary, there are signs that methodological advancements have been made much faster than progress has been made with respect to full-scale validation. In future work, neuro-oncology AI researchers need to move away from focusing exclusively on accuracy and embrace other measures such as calibration, quantification of uncertainty, evaluation of clinical impact, and external validation.

### 5.5 Trustworthiness and clinical validation gap

In addition to being effective predictors, trustworthy AI needs to be reliable, well-calibrated, robust, and clinically useful. Yet, the evaluation of the reviewed papers shows an obvious disparity between performance-related and trustworthiness-related approaches to assessing these models.

As seen from Table 19, traditional performance indicators like accuracy, Dice, sensitivity, specificity, and F1-scores prevail in research; calibration indicators, metrics related to uncertainty estimation, and clinical usefulness criteria are mentioned quite rarely. Calibration measures like Brier Score, Expected Calibration Error (ECE), Maximum Calibration Error (MCE), and Negative Log-Likelihood (NLL) can be found in less than 7% of the reviewed works. This problem is especially pertinent since the accuracy of a model does not imply its trustworthiness. A system can have great benchmark scores but at the same time can have poor-calibrated confidences or be unreliable in distribution shift situations. Thus, performance scores alone are not enough for assessing the clinical readiness of a model. Fortunately, some promising recent works started to use the analysis of the calibration, uncertainty quantification, external validation cohorts, and decision-curve analysis which shows that there is already a move toward the trustworthy evaluation of AI. However, these approaches still are exceptions rather than the rule.

**Table 19:** Imbalance between performance-oriented and trustworthiness-oriented evaluation.

Evaluation dimension	Frequency (%)
Accuracy-based Performance Metrics (ACC)	73.8
Diagnostic Metrics (AUC)	24.6
Calibration metrics	6.6
Clinical utility metrics	3.3
Uncertainty metrics	1.6

Percentages are calculated using the same 61-study evaluation subset described in Section 5.4. Evaluation dimensions are not mutually exclusive because individual studies may report multiple evaluation measures.

Overall, these results expose an underlying imbalance within modern neuro-oncology AI research. As is clear from the extensive use and reporting of performance metrics, there has been far less focus on developing reliable evaluation methodologies. Thus, many state-of-the-art algorithms show excellent performance metrics but lack adequate validation for their calibration, uncertainty handling, robustness to distributional shifts, or practical applicability in clinical settings. It is therefore imperative that future research efforts emphasize both innovative validation methods along with algorithm design.

### 5.6 Synthesis of findings against the research questions

The findings of this systematic review provide direct evidence addressing the three research questions. For RQ1, the reviewed literature demonstrates a clear progression from conventional deep learning and CNN-based approaches toward explainable AI, advanced architectures such as Vision Transformers and Mamba, privacy-preserving learning, and multimodal MRI integration. For RQ2, explainability is substantially more represented than privacy-preserving and federated approaches, while the integration of XAI and FL within a unified brain tumor MRI framework remains limited. The reviewed literature therefore indicates a gap between the development of interpretable models and privacy-preserving multi-institutional learning. For RQ3, the review identifies three overarching gaps: insufficient generalizability and clinical validation, limited quantitative validation of explanations and trustworthiness measures, and deployment constraints arising from heterogeneous data, privacy-preserving learning, and computational burden. These issues are examined in Sections 5.1–5.5 and consolidated into the open challenges and future priorities presented in Sections 6 and 7.

### 5.7 Emerging research priorities and strategic directions

From the review's overall findings, several research directions can be prioritized in the future in order to facilitate the development of trustworthy AI models for brain tumor detection:

1. Validation of AI Models in Heterogeneous and Multi-Institutional Environments: In the coming years, validation studies need to focus more on evaluating AI models based on large and heterogeneous datasets that come from different institutions, instead of limiting themselves to benchmark databases.
2. Development of Quantitative and Grounded XAI Systems: The future research related to XAI needs to go further from visualizing explanations to validating quantitative explanations. These future models will need to include uncertainty estimates, agreement with clinicians, causality attribution, and biological grounds.
3. Adaptive and non-IID resilient Federated Learning: The research field of privacy-preserving learning should shift from simple FedAvg approaches to more advanced, communication-efficient and adaptive Federated Learning that can deal with missing modality data, severe class imbalance, and heterogenous scanner settings.
4. Multi-omics and multimodal reasoning engines: Multimodal learning architectures should unify all information obtained from images, radiomic data, genomic, pathological, and clinical data into reasoning pipelines. Nonetheless, these methods have to take into account computational efficiency, calibration, and interpretability issues.
5. Unified benchmarking of trustworthiness: It is highly necessary to develop standardized benchmarking protocols that would cover all aspects of trustworthiness assessment in the context of AI applications to brain tumor detection. In addition to accuracy, future frameworks should be evaluated based on their performance in terms of explanation, uncertainty handling, fairness, computational efficiency, and repeatability.

In order to consolidate the results of this review, Table 20 demonstrates a thematic synthesis of selected collaborative and multimodal AI models designed for brain tumor detection. The table provides an overview of the fundamental structure used in the framework of each research paper, the major metrics used to demonstrate the performance of these models, the nature of datasets used, the methods of interpretability used in each case, and a critique of the reliability of these measures.

**Table 20:** Thematic synthesis of performance, interpretability, and reliability across collaborative and multimodal frameworks.

Core architecture	Key metrics	Size (*n*)	Interpretability/tool	Critical analysis/reliability
Multi-modal CNN + ChatGPT interface (Rawas and Samala, 2024)	High (Qual.)	MRI	Linguistic Explanations	Hallucination risk: LLM false narratives.
RCSF-CNN (spatial fusion) (Kesav and Rajini, 2024)	98.99% Acc.	**32**	Class Activation Map	Small data: Dubious reliability.
FL + lesion delineation (Lee et al., 2024)	0.78 Dice	Multi	Decentralized Delineation	Comparable to centralized models.
ResNet-18 + FL (Anoosha and Seetharamulu, 2025)	98.00% Acc.	MRI	Local weight updates	Institutional data sovereignty.
AlexNet, LayerCAM, BiLSTM (Pathan et al., 2025)	99.56% Acc.	BraTS	LayerCAM / BiLSTM	High training acc; needs k-fold.

Concluding, it becomes apparent that the future of brain tumor imaging is less likely to be about reaching higher accuracy rates than about creating explainable, privacy-preserving, biologically interpretable, efficient, and clinically relevant AI systems.

## 6 Open challenges

However, despite all the advances in brain tumor detection research that can be considered trustworthy, there is still a set of crucial obstacles impeding the reliable implementation of such technologies in practice. For instance, one of the major drawbacks of neuro-oncology AI is its high dependence on benchmark datasets such as BraTS, TCGA, TCIA, and Figshare. As a result, models trained on such datasets may demonstrate their inefficiency in real-life settings due to lack of generalization. Furthermore, despite the availability of explainable AI technologies (Grad-CAM, SHAP, and saliency mapping), the biologically causal explanation and physician-oriented assessment of AI frameworks remain problematic.

As demonstrated in Figure 13, current limitations associated with trustworthiness in brain tumor detection using AI go beyond merely being linked to predictive capabilities and include several interlinked challenges pertaining to dependency on datasets, reliability of explanation methods, scalable computation, robust Federated Learning, variability in images, and translating regulations. These limitations illustrate how the aforementioned challenges prevent the experimental implementation of AI from becoming practical in neuro-oncology. Despite the presence of external testing and some multi-institutional evaluations, prospective multi-center clinical validation and systematic clinician-in-the-loop assessment remain limited. In particular, independent clinical testing, radiologist comparison, prospective evaluation, and workflow-based assessment are not yet consistently incorporated into the validation of brain tumor AI systems. Another important issue relates to the growing complexity of architectures in multimodal, transformer, and optimization approaches. Although such an approach allows improving the performance of classifiers and predictions, it is frequently accompanied by the problems of heavy computation load, lack of reproducibility, and challenges in implementation due to the limited resources in healthcare institutions. Additionally, Federated Learning approaches that ensure privacy suffer from the presence of non-independent and identically distributed datasets, communication overhead, and poor results achieved under strict privacy conditions. Issues regarding missing modalities, artifacts, scanner heterogeneity, calibration of uncertainty, and robustness to domain shift have been largely ignored by existing neuro-oncology artificial intelligence models. Moreover, issues related to regulatory approval, the lack of prospective multicenter validation, and clinician-in-the-loop framework for evaluation persist.

**Figure 13:**
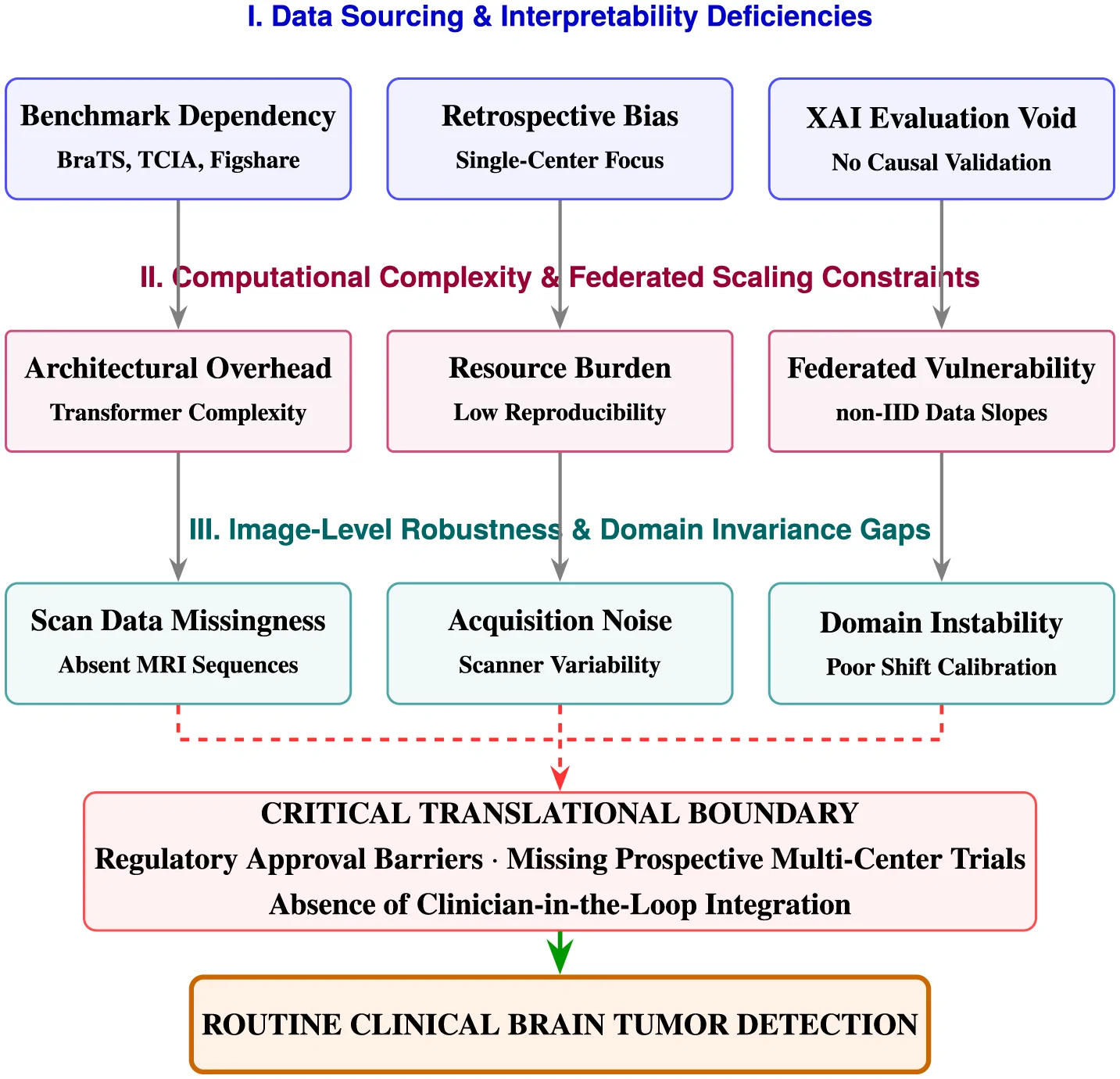
Global open challenges limiting the clinical translation of trustworthy brain tumor detection using AI systems. The figure summarizes major challenges associated with dataset dependency, retrospective bias, explainability validation, computational complexity, Federated Learning scalability, imaging variability, domain shifts, and regulatory deployment barriers affecting routine clinical brain tumor detection.

### 6.1 Limitation

While the current review gives an overview of the progress made in analyzing brain tumors using MRI images through artificial intelligence techniques, there are some limitations exists. First, the literature search was conducted exclusively in Scopus and was restricted to English-language publications. The use of a single bibliographic database and a TITLE-ABS-KEY search strategy may have resulted in the omission of relevant studies indexed in other databases, such as PubMed/MEDLINE, Web of Science, IEEE Xplore, or Embase. In addition, studies that do not explicitly mention explainability, interpretability, Federated Learning, or privacy in their titles or abstracts may not have been retrieved. Formal inter-rater reliability was not measured; therefore, some assessor subjectivity cannot be completely excluded. Accordingly, the findings should be interpreted as a focused systematic synthesis of trustworthy-AI-related brain tumor MRI research rather than an exhaustive representation of the entire brain tumor AI literature. Secondly, owing to the rapid development of artificial intelligence, some new approaches might have appeared after the beginning of the review process and, therefore, have not been considered in this study. Finally, owing to great variability in terms of data sets, image acquisition techniques, metrics used, and experimental methodology among the articles analyzed, a quantitative meta-analysis could not be carried out.

## 7 Future directions

In terms of trustworthy brain tumor detection using AI, future research directions include improving the clinical utility, robustness, and biological plausibility of DL methods. The potential approach that could address these needs is developing the concept of radiology foundation models using large pre-trained models based on multi-center MRI data sets to reduce the reliance on benchmark datasets, like BraTS. Moreover, combining Explainable AI and Federated Learning approaches can provide an effective privacy-preserving way of distributed learning while preserving clinically useful explanations of the decision process. Multimodal and radiogenomic intelligence can be considered as another key aspect of future developments as they may allow analyzing MRI, histopathology, genomic features, and clinical data simultaneously in order to provide personalized predictions of disease progression. Future work should also aim at developing and applying rigorous trustworthiness evaluation protocols including the analysis of predictive uncertainty, adversarial robustness, calibration, and clinician-centered explainability. Additionally, it might be worthwhile exploring lightweight architectures and continual learning approaches as well as developing hybrid models based on transformers and other DL approaches.

## 8 Conclusion

This systematic review synthesized 146 studies published between 2020 and 2026 to examine the evolution of trustworthy AI for brain tumor MRI analysis across five primary research themes: Explainable AI (XAI), Deep Learning Frameworks, Privacy and Federated Learning, Advanced Architectures (Transformers/Mamba), and Multi-modal MRI Integration. The quantitative thematic analysis showed that Explainable AI (XAI) was the most represented research theme, with 55 studies (37.7%), followed by deep learning frameworks with 51 studies (34.9%). Privacy and Federated Learning were represented in 20 studies (13.7%), while advanced architectures based on Transformers and Mamba accounted for 15 studies (10.3%). Multi-modal integration was represented in 5 studies (3.4%). Each study was assigned to one primary research theme according to its dominant methodological focus, and the five categories therefore collectively represent all 146 included studies. These findings indicate that explainability and conventional deep learning frameworks constitute the largest thematic groups in the reviewed literature, whereas privacy-preserving, advanced-architecture, and multimodal studies represent smaller primary thematic groups.

The publication trend further demonstrated a substantial concentration of recent research. The reviewed studies comprised 2 publications in 2020, 2 in 2021, 9 in 2022, 11 in 2023, 31 in 2024, 77 in 2025, and 14 in 2026. Thus, 122 of the 146 included studies (83.6%) were published during 2024–2026, indicating a marked recent expansion of research on trustworthy AI for brain tumor MRI analysis. Dataset analysis also revealed substantial dependence on established benchmark resources. BraTS was the most frequently reported dataset, occurring in 63 of the 146 included studies (43.2%). Because individual studies could report multiple datasets, dataset frequencies were not mutually exclusive and were therefore not expected to sum to 146. The dataset–task analysis further showed that dataset utilization varied across segmentation, classification, prognosis, and molecular characterization tasks. For these analyses, the individual study was used as the counting unit; within each task category, a study was counted once for each distinct dataset it reported, irrespective of repeated use of that dataset across experiments. Studies addressing multiple tasks were counted in each applicable task category, and therefore task-specific frequencies and denominators were not mutually exclusive. The review also identified an imbalance between conventional performance evaluation and trustworthiness-oriented evaluation. Accuracy-based metrics were reported in 73.8% of the representative studies analyzed, while F1-score and Dice-based measures were reported in 27.9% each. In contrast, calibration, clinical utility, and uncertainty measures were comparatively uncommon, reported in 6.6%, 3.3%, and 1.6% of the representative studies, respectively. This quantitative evidence indicates that predictive performance remains substantially more established than systematic evaluation of calibration, uncertainty, and clinical usefulness. Overall, the reviewed evidence demonstrates measurable progress toward trustworthy AI for brain tumor MRI analysis, particularly in explainability and the adoption of emerging architectures. However, the relatively limited representation of Federated Learning, multimodal integration, and trustworthiness-oriented evaluation, together with dependence on benchmark datasets, highlights persistent gaps in privacy-preserving collaboration, external validation, clinical validation, and robust assessment. These findings support the need for future studies to complement predictive performance with externally validated, clinically grounded, and quantitatively assessable measures of trustworthiness.
